# Integrating digital gait data with metabolomics and clinical data to predict outcomes in Parkinson’s disease

**DOI:** 10.1038/s41746-024-01236-z

**Published:** 2024-09-06

**Authors:** Cyril Brzenczek, Quentin Klopfenstein, Tom Hähnel, Stefano Sapienza, Jochen Klucken, Holger Fröhlich, Enrico Glaab

**Affiliations:** 1 Biomedical Data Science Group, Luxembourg Centre for Systems Biomedicine (LCSB), University of Luxembourg, Esch-sur-Alzette, Luxembourg; 2 Department of Bioinformatics, Fraunhofer Institute for Algorithms and Scientific Computing, Sankt Augustin, Germany; 3 Department of Neurology, Medical Faculty and University Hospital Carl Gustav Carus, TUD Dresden University of Technology, Dresden, Germany; 4 Luxembourg Centre for Systems Biomedicine, University of Luxembourg, Esch-sur-Alzette, Luxembourg; 5 Luxembourg Institute of Health, Strassen, Luxembourg; 6 Centre Hospitalier de Luxembourg, Strassen, Luxembourg; 7 Bonn-Aachen International Center for IT (b-it), University of Bonn, Bonn, Germany; 8 Centre Hospitalier Emile Mayrisch, Esch-sur-Alzette, Luxembourg; 9 Laboratoire National de Santé, Dudelange, Luxembourg; 10 Association of Physiotherapists in Parkinson’s Disease Europe, Esch-sur-Alzette, Luxembourg; 11 Faculty of Science, Technology and Medicine, University of Luxembourg, Esch-sur-Alzette, Luxembourg; 12 Department of Epidemiology, CAPHRI School for Public Health and Primary Care, Maastricht University Medical Centre, Maastricht, the Netherlands; 13 Private Practice, Ettelbruck, Luxembourg; 14 Parkinson Luxembourg Association, Leudelange, Luxembourg; 15 Luxembourg Center of Neuropathology, Dudelange, Luxembourg; 16 Department of Life Sciences and Medicine, University of Luxembourg, Esch-sur-Alzette, Luxembourg; 17 Private Practice, Luxembourg, Luxembourg

**Keywords:** Parkinson's disease, Diagnostic markers

## Abstract

Parkinson’s disease (PD) presents diverse symptoms and comorbidities, complicating its diagnosis and management. The primary objective of this cross-sectional, monocentric study was to assess digital gait sensor data’s utility for monitoring and diagnosis of motor and gait impairment in PD. As a secondary objective, for the more challenging tasks of detecting comorbidities, non-motor outcomes, and disease progression subgroups, we evaluated for the first time the integration of digital markers with metabolomics and clinical data. Using shoe-attached digital sensors, we collected gait measurements from 162 patients and 129 controls in a single visit. Machine learning models showed significant diagnostic power, with AUC scores of 83–92% for PD vs. control and up to 75% for motor severity classification. Integrating gait data with metabolomics and clinical data improved predictions for challenging-to-detect comorbidities such as hallucinations. Overall, this approach using digital biomarkers and multimodal data integration can assist in objective disease monitoring, diagnosis, and comorbidity detection.

## Introduction

Parkinson’s disease (PD) exhibits a remarkable heterogeneity in its clinical manifestations, covering a spectrum of motor and non-motor symptoms that vary widely among patients^1,2^. This diversity poses significant challenges to the accurate diagnosis of PD and the prognosis of individual disease progression, making effective management and therapy development difficult^3^. Each patient may experience a different pattern of motor and non-motor symptoms at disease onset, as well as different patterns of disease progression. Therefore, careful and time-consuming assessment is required to capture the individual spectrum of symptoms and subsequently allow for tailored interventions to manage individual symptomatology and improve quality of life. Improving the understanding of the varied manifestations of PD could guide the development of future therapies and lead to more personalized medicine strategies that can better address the unique needs of each patient and thereby achieve improved effectiveness across the broad spectrum of PD phenotypes.

Digital biomarkers are defined as characteristics collected from digital health technologies that are measured as indicators of normal biological processes, pathogenic processes, or responses to an exposure or intervention, including therapeutic interventions^4^. These markers represent a new opportunity in the diagnosis and management of PD, providing objective, quantifiable physiological and behavioral data collected through digital devices such as sensors and mobile applications. An example of such data is gait sensor data, which captures detailed walking patterns and can reveal subtle motor impairments not easily detected by conventional clinical assessments. Compared to traditional clinical measurements, digital biomarkers offer several potential advantages: they are less invasive, can be collected continuously over time, and may reduce patient burden by allowing measurements to be taken at home, minimizing the need for frequent clinical visits^5^. This continuous monitoring capability has the potential to facilitate a real-time understanding of disease progression and patient response to treatment. By integrating digital biomarkers into the diagnostic and prognostic processes in healthcare, clinicians could achieve a more detailed view of PD variability, with the potential to improve the precision of outcome predictions and enabling more personalized disease management plans. This approach may help to address or alleviate some of the current limitations in predicting outcomes for PD by harnessing advanced technologies to gather more objective, comprehensive and accurate data on the heterogeneous and sometimes transitory impairments in PD.

Previous studies on digital biomarkers for PD, particularly those focusing on gait-related characteristics, have already highlighted the potential of wearable technology and continuous monitoring to improve diagnosis and management of the disease. For instance, Shah et al. identified key gait-related digital biomarkers such as turn angle and swing time variability as significant discriminators of mobility between PD patients and healthy controls during a week of continuous monitoring^6^. A similar investigation by Rehman et al. emphasized the importance of combining classical spatiotemporal features with signal processing-based gait characteristics, which provided higher classification accuracy, and highlighted their potential for early disease identification^7^. In addition, information theory derived measures have also been applied successfully, e.g., Coates et al. studied the sample entropy of digital gait data as a biomarker, showing its effectiveness in reflecting changes in gait regularity over time among PD patients^8^. These prior findings collectively support the use of digital gait-related biomarkers as informative indicators of motor impairment and other disease severity and diagnostic outcomes in PD, providing a potential pathway for improved clinical assessments and possibly earlier and more effective therapeutic interventions.

To complement previous work, this cross-sectional, monocentric, and observational study compared PD patients with controls to assess the predictability of the current diagnostic status and motor score impairment from a brief digital gait-based assessment performed during a single clinical visit per person. In addition, we compared patients with respect to their current gait and mobility impairments, comorbidities, and motor score progression rates to assess the predictability of these important characteristics for disease monitoring. This was done in two ways: (a) Using digital gait data alone (with a focus on detecting the following outcomes that are strongly related to gait alteration: PD vs. control diagnostic status, motor score outcomes, and mobility and gait impairment outcomes), and (b) through its integration with metabolomics and simple clinical descriptors (with a focus on detecting the following outcomes that are not closely related to gait changes and are more challenging to predict using only a single data modality: comorbidities, non-motor scores, and motor score progression rates), an aspect that, to our knowledge, has not been previously explored in the literature. The aim of these analyses was to determine whether automated gait measurements during a brief walking exercise, lasting at most a few minutes, or their combination with a few selected metabolite and clinical features, could provide more objective and less time-consuming to obtain surrogate biomarker signatures for multiple PD monitoring outcomes that typically require extensive and burdensome clinical examinations.

**Fig. 1:**
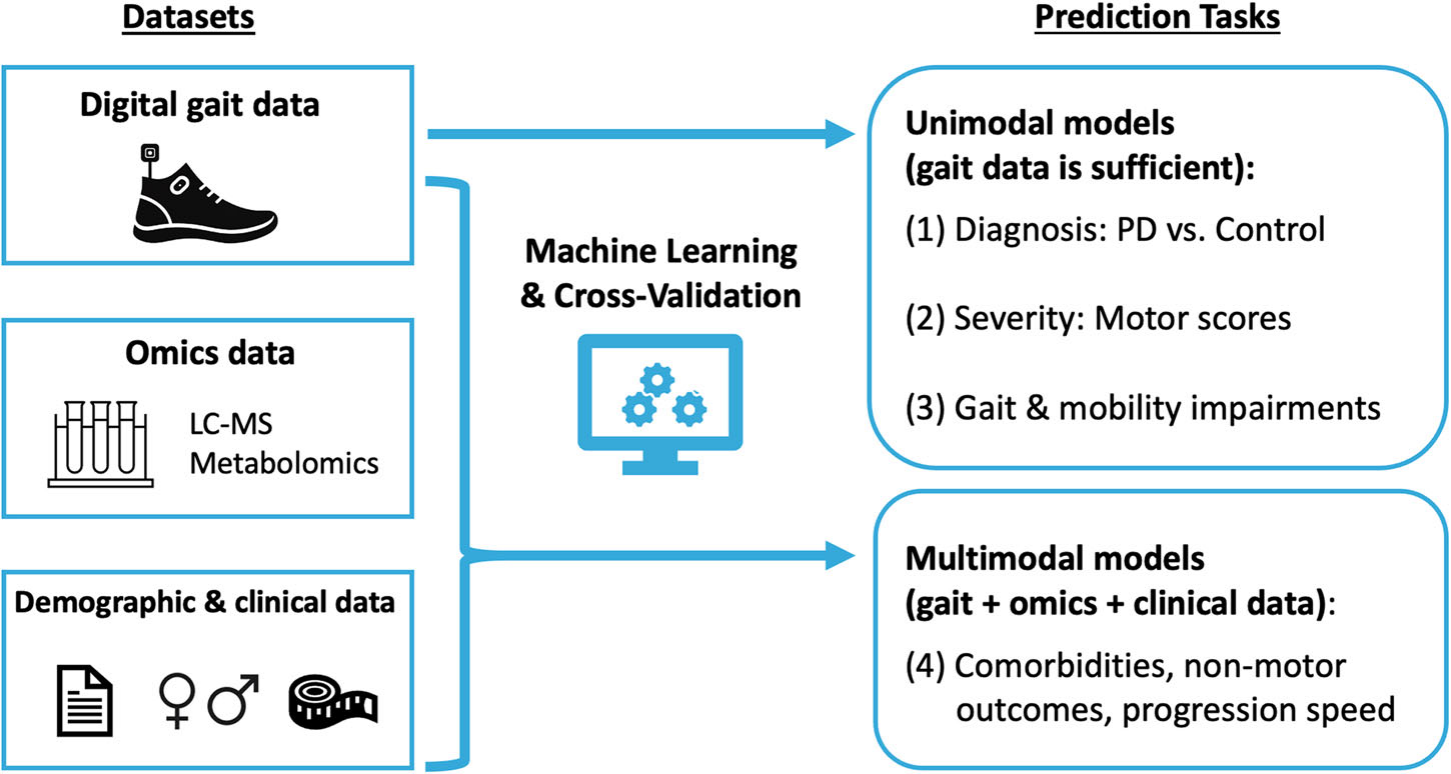
Schematic overview of the study workflow. The input data types used for the cross-validated machine learning analyses are highlighted on the left and the prediction tasks on the right. The prediction tasks focus on the estimation of four groups of outcomes (roughly sorted by increasing complexity): (1) disease diagnosis, (2) motor score severity, (3) gait and mobility impairments, and (4) comorbidities, non-motor outcomes, and progression rate (measured by the average annual change in the MDS-UPDRS III motor score over four years of follow-up and categorized as slow or fast, depending on whether the change falls in the lower or upper quartile, respectively). For the first three data modalities, unimodal machine learning models were built using gait data only, as this was sufficient to achieve satisfactory cross-validation performance, whereas for the more challenging fourth group of tasks, aimed at detecting comorbidities, non-motor outcomes and progression rate subgroups, multimodal models combining gait, omics and clinical data were built in addition to comparing the individual data modalities.

Using the gait sensor technology eGaIT (embedded Gait Analysis using Intelligent Technologies)^9^, gait data was previously collected from a selection of 291 subjects, including 162 PD patients and 129 controls, from the Luxembourg Parkinson’s Study^10^. Data was captured through standardized tasks that ranged from simple walking to complex multitasking scenarios. This approach allowed for the extraction of precise gait features, which were then analyzed alongside omics data and clinical assessments. We tested different types of machine learning approaches, including Stochastic Gradient Boosting, Support Vector Machines and Random Forest, among others, to build, cross-validate and interpret predictive models. Our analyses aimed to explore multiple health outcomes in PD, namely to (i) distinguish between PD patients and control subjects, (ii) assess current disease severity in terms of motor scores, (iii) identify specific gait impairments such as freezing of gait, and (iv) detect common non-motor symptoms and comorbidities in PD, such as cognitive impairment, as well as patient subgroups with different rates of motor score progression. Since comorbidities and non-motor symptoms, such as cognitive impairment and depression, are not directly related to changes to motor changes in PD, but may still influence gait patterns—given that gait involves both cognitive and motor functions—it is valuable to investigate whether these more difficult-to-detect outcomes can be more effectively assessed using multimodal data to improve machine learning models. This also applies to the classification of motor score progression rates, which can be assessed more comprehensively by considering current metabolic and clinical changes, in addition to changes in gait characteristics. Therefore, while we focused on gait data as input to predict the first three categories of outcomes strongly associated with motor impairment, we compared and integrated digital gait biomarkers with clinical and metabolomics data derived from the baseline visit for the same subjects (see detailed descriptions of these datasets in the “Methods” section) for the more difficult-to-assess outcome variables related to comorbidities, non-motor symptoms and progression rates (see also the workflow overview in Fig. 1). This comprehensive analysis clearly distinguishes our work from previously published papers on digital gait assessments, which typically focused on the discrimination of PD patients versus control subjects^6,11–13^. Our study was designed to assess the strengths and weaknesses of each individual data type and explore the potential synergies achieved through integrative predictions, an aspect that we regard as novel compared to prior work in the literature. The integrative approach enhanced the robustness of our predictions, and for some of the considered PD outcomes, it also led to marked improvements in accuracy. In addition, the analyses of the most predictive attributes provided insights into the informative value of specific biomarker features, as well as the complex interplay between different types of biomarkers – an aspect that has largely been neglected so far in the existing literature on digital gait assessments.

Overall, the comparative and integrative analyses of digital gait biomarkers with clinical and metabolomics data presented here underscore both the utility of the gait data and the potential benefits of combining multiple complementary data sources for PD research. For multiple clinically relevant outcomes in PD, including the PD vs. control diagnostic status, the MDS-UPDRS III motor score, assessment of gait and mobility impairment, and the occurrence of multiple comorbidities, our results show that either the digital gait data alone provide significant predictive information (particularly for the diagnostic status and the motor and gait-related outcomes), or that the integration of gait, clinical and metabolomics data can improve the diagnostic accuracy compared to using only single data modalities. Taken together, these strategies for using digital gait markers or multiple data modalities for machine learning prediction in PD could help to pave the way for a more objective, standardized, and largely automated disease monitoring.

## Results

### PD vs. control classification

To evaluate the efficacy of digital gait biomarkers for detecting disease associated gait impairments, we initially focused on PD vs. control classification. We compared two types of input features derived from gait sensor data: extracted gait parameters and raw signal time series features (see “Methods” section “Data collection”). Each feature set was analyzed using different machine learning models to determine their diagnostic potential within a 10-fold cross-validation (CV) framework (see “Methods” section on “Machine learning, cross-validation, and model interpretation”).

#### Results from extracted gait parameters

Using extracted gait parameters, the 10-fold CV results for discriminating PD from controls (Table 1, second row) showed median AUC scores between 65% and 77%, depending on the model. The Deep Boosting (DEEP) model achieved the highest median AUC at 76.9%, suggesting that decision tree-based ensemble methods are well suited for the considered heterogeneous data. Demographic covariates alone (age, sex) provided considerably lower predictive accuracy (median AUC of 59.1%), indicating that digital gait parameters add substantial information.

**Table 1:** Cross-validated predictive performance for PD vs. control classification

Models		Linear SVM	RBF SVM	RF	Stochastic GBM	DEEP	XGB	Clinical confounders (median)
10-fold CV AUC median (mad)	Extracted gait parameters	0.649 (0.13)	0.695 (0.18)	0.743 (0.11)	0.737 (0.12)	^a^0.769 (0.07)	0.754 (0.06)	0.591 (0.09)
	Raw signal time series features	0.847 (0.04)	0.865 (0.08)	0.834 (0.05)	0.876 (0.09)	0.910 (0.06)	^a^0.917 (0.07)	

Cross-validated predictive performance for PD vs. control classification using different machine learning methods and extracted gait parameters or time series features computed from the raw gait signal data as input “Clinical confounders” refers to a model that was solely trained with age and sex as predictors and serves as a comparator.

*AUC* area under the Receiver Operating Characteristic Curve, median and median absolute deviation (mad) across 10 cross-validation (CV) cycles, *SVM* support vector machine, *RBF* radial basis function, *RF* random forest, *DEEP* deep boosting, *XGB* extreme gradient boosting, *GBM* gradient boosting machines.

^a^The highest median AUC for each row.

#### Results from raw signal time series features

When using time series features derived from the raw signal data, AUC scores ranged from 83% to 92% (Table 1, bottom row). The extreme gradient boosting (XGB) and Deep Boosting (DEEP) models had the highest median AUC scores with 91.7% and 91.0%, respectively. These improvements over gait-specific parameters highlight the utility of using time series features for capturing detailed gait characteristics indicative of PD. Although these features are complex to interpret, and manually crafted gait features may be preferred for explainable models, the cross-validation results show that using features more closely reflecting the raw signal data improves diagnostic performance. This suggests that using time series features to analyze the full complexity of gait dynamics is more informative for PD symptom detection than relying on a limited set of extracted gait features. This may be due to more comprehensive data coverage and finer granularity of the time series features, reflecting subtle gait abnormalities not apparent in processed gait parameters.

### Severity prediction: PD motor score estimation/surrogate biomarker modeling

To complement PD vs. control diagnostic predictions with a machine learning assessment of motor severity, we performed predictive modeling of PD motor severity using the MDS-UPDRS Part III sum score^14^ at the same visit as a target variable. Our goal was to assess the utility of gait-specific features as potential digital surrogate biomarkers for commonly used motor performance assessments. Successful validation of gait features could reduce costs, time, and effort in traditional clinical exams, allowing for streamlined and potentially at-home assessments.

We applied the same cross-validated classification pipeline used for diagnostic predictions to the motor score analyses, binarizing UPDRS Part III total scores using a median threshold (19 points) and predicting whether patients fall into the low or high total score category.

Similar to PD vs. control classification, tree-based ensemble methods showed superior diagnostic performance. Using time series features from raw gait data, the Random Forest model achieved the highest median AUC at 75.4%, which was remarkably above the performance achieved when only using age and sex as predictors (55.4%). For most modeling approaches, using time series features from raw gait data provided higher average AUC scores than gait-specific features (Table 2, second row).

**Table 2:** Cross-validated predictive performance for MDS-UPDRS Part III classification

Models		Linear SVM	RBF SVM	RF	Stochastic GBM	DEEP	XGB	Clinical confounders (median)
10-fold CV AUC median (mad)	Extracted gait parameters	0.610 (0.21)	0.654 (0.09)	0.705 (0.12)	0.615 (0.16)	0.667 (0.12)	^a^0.76 (0.12)	0.554 (0.12)
	Raw signal time series features	0.664 (0.09)	0.666 (0.08)	^a^0.754 (0.22)	0.655 (0.13)	0.740 (0.10)	0.745 (0.06)	

Cross-validated predictive performance for MDS-UPDRS Part III classification of whether MDS-UPDRS Part III sum scores are above or below the media score using different machine learning methods and time series features computed from either the extracted gait parameters or the raw gait signal data as input. “Clinical confounders” refers to a model that was solely trained with age and sex as predictors and serves as a comparator.

*AUC* area under the Receiver Operating Characteristic Curve, median and median absolute deviation across 10 cross-validation (CV) cycles, *SVM* support vector machine, *RBF* radial basis function, *RF* random forest, *DEEP* deep boosting, *XGB* extreme gradient boosting, *GBM* gradient boosting machines.

^a^The highest median AUC for each row.

**Fig. 2:**
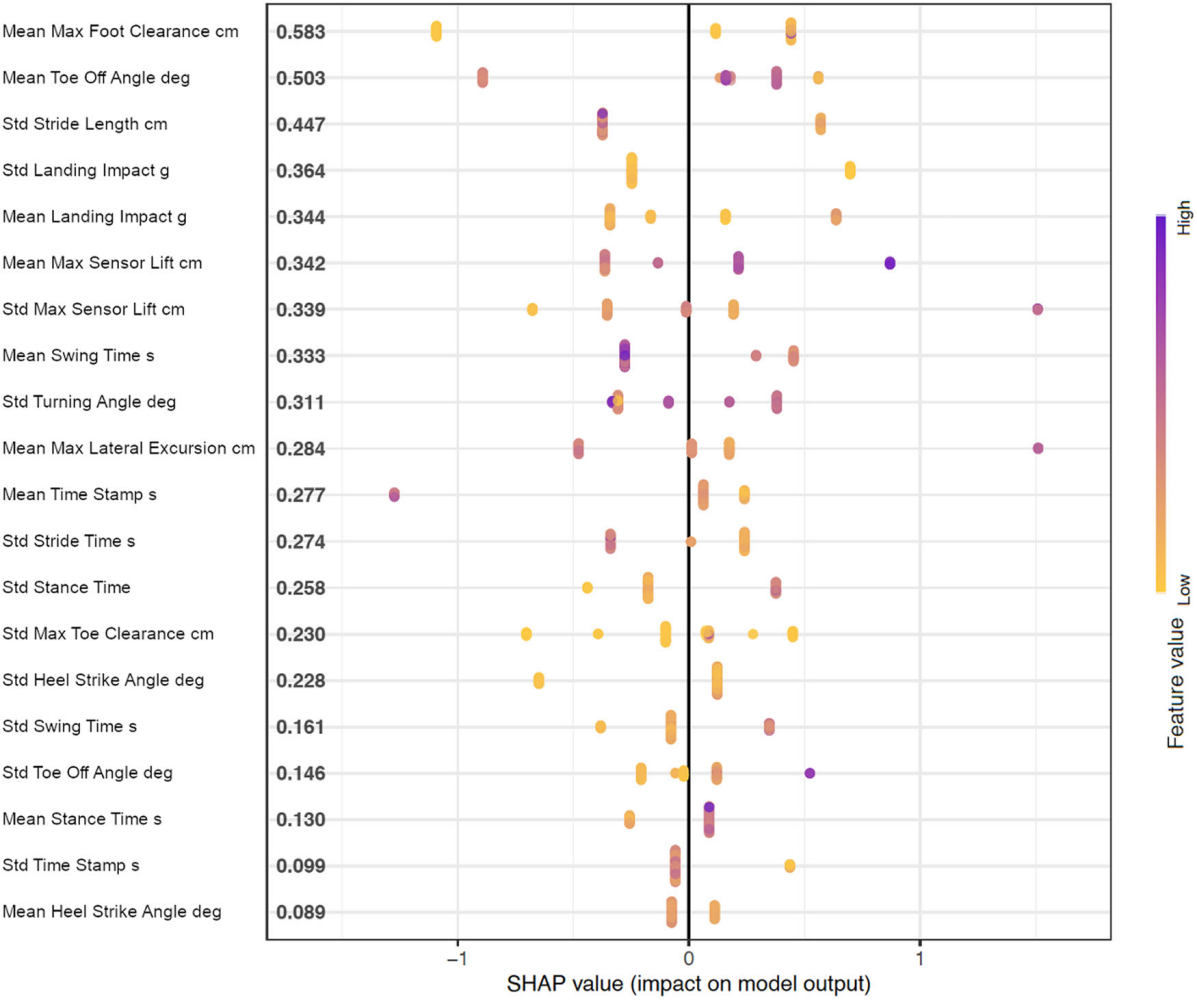
SHAP value plot of the top-ranked features for predicting low vs. high UPDRS 3 motor score outcomes. The plot shows the gait-specific digital biomarker features with the highest SHAP values for predicting low vs. high UPDRS 3 motor score outcomes using extreme gradient boosting for machine learning. The color coding from purple to yellow represents the feature value range from low to high. The labels on the left correspond to the individual gait features that were most predictive in terms of the absolute SHAP value, sorted from top to bottom (corresponding absolute SHAP values are shown in bold on the left side of the plot; for a description of the individual features see Supplementary Table 1).

As the machine learning analysis can help to identify which features are most informative to detect motor severity in PD, we conducted a SHAP value analysis^15^ for the more interpretable model using gait parameters as input. The SHAP value plot in Fig. 2 shows the importance of individual gait features in predicting motor scores using extreme gradient boosting, the best-performing technique in cross-validation. SHAP values indicate how each feature shifts the model output from the baseline prediction. The mean values for the gait parameter “Maximum Foot Clearance” (corresponding to the maximum elevation of the foot from the ground during the swing phase) and “Toe Off Angle” (corresponding to the angle between the heel and the surface at the beginning of the swing phase) had the highest SHAP values, likely reflecting a greater degree of foot clearance difficulty in patients with severe motor impairments. Other important features included the standard deviation of “Stride Length” and “Landing Impact,” indicating increased variation in these parameters with more severe motor impairments. These results demonstrate the potential of digital gait sensor data combined with machine learning, especially tree-based methods, for classifying motor severity in PD. This approach could enable simple at-home gait-based assessments as surrogate biomarkers for motor performance, reducing the need for more labor-intensive clinical evaluations. In addition, the SHAP analysis provides insights into the most influential features for predicting UPDRS 3 motor outcomes, helping clinicians and researchers to prioritize specific gait parameters in routine evaluations of disease severity.

### Prediction of gait- and mobility-related outcomes

The analysis of gait- and mobility-related impairments in PD was a key focus of this study, as digital gait biomarker data is expected to be highly informative for classifying gait and movement-associated outcomes. We aimed to detect three main outcomes: (1) *Freezing of gait (FoG)*, using the FoG severity score from the FOGQ questionnaire^16^, (2) the *mobility sub-score* from the PDQ39 quality-of-life questionnaire^17^, and (3) the general *occurrence of gait disorders* as part of standard clinical assessments in the Luxembourg Parkinson’s Study^10^. For the quantitative outcomes (FOGQ score and PDQ39 mobility sub-score), we binarized the scores into values above and below the median to apply classification approaches and performance metrics comparable to those used for categorical outcomes. As the focus of these analyses was on maximizing model predictivity rather than interpretability, we used the comprehensive set of time series features from raw gait data for the machine learning analyses, applying the same CV framework and machine learning approaches as for the PD vs. control and motor severity predictions.

#### Prediction of freezing of gait (FoG)

Consistent with previous analyses, a tree-based ensemble learning method (stochastic GBM) led to the highest median AUC of 91.7%, which was remarkably above the 60.9% achieved when only using age and sex (clinical confounders) as predictors (see Table 3, first data row). These results highlight the significant predictive performance of gait-based machine learning for FoG score classification.

**Table 3:** Cross-validated performance for three motor performance-related classification problems

Models		Linear SVM	RBF SVM	RF	Stochastic GBM	DEEP	XGB	Clinical confounders (median)
10-fold CV AUC median (mad)	FoG	0.694 (0.04)	0.792 (0.19)	0.889 (0.11)	^a^0.917 (0.12)	0.833 (0.12)	0.903 (0.14)	0.609 (0.11)
	Gait disorder occurrence	^a^0.742 (0.14)	0.562 (0.20)	0.589 (0.11)	0.569 (0.18)	0.577 (0.17)	0.652 (0.22)	0.554 (0.13)
	PDQ-39 mobility sub-score	0.646 (0.12)	0.781 (0.09)	0.759 (0.19)	0.795 (0.16)	^a^0.799 (0.14)	0.764 (0.12)	0.535 (0.09)

Cross-validated performance for three classification problems using different machine learning methods and time series features computed from the raw gait signal data as input: (1) Freezing of Gait (FoG) score binary classification into scores above/below the median; (2) gait disorder occurrence prediction (yes/no); (3) predicting whether the PDQ-39 mobility sub-score of PD patients is above the median in the cohort (yes/no). “Clinical confounders” refers to a model that was solely trained with age and sex as predictors and serves as a comparator.

*AUC* area under the Receiver Operating Characteristic Curve, median and median absolute deviation (mad) across 10 cross-validation (CV) cycles, *SVM* support vector machine, *RBF* radial basis function, *DEEP* deep boosting, *XGB* extreme gradient boosting, *RF* random forest, *GBM* gradient boosting machines.

^a^The highest median AUC for each row.

#### Gait disorder occurrence detection

For inferring the presence of general gait disorders in PD as diagnosed by the treating neurologist, the 10-fold CV framework revealed varying model performances (Table 3, second data row). The Linear SVM model achieved the highest median AUC at 74.2%, again with a remarkable difference to the 55.4% achieved by the “clinical confounders” model.

#### Prediction of mobility scores

While digital sensor data mainly captures gait-related changes in PD, we investigated if it could also predict overall mobility impairments using the Parkinson’s Disease Questionnaire-39 (PDQ-39) mobility sub-score. In the cross-validation, the deep boosting model (DEEP) achieved the highest median AUC of 79.9%, which was remarkably above the 53.5% achieved only with age and sex as predictors (Table 3, third data row).

### Detection of comorbidities, non-motor symptoms and disease progression sub-groups

PD is often associated with various comorbidities that affect patients’ quality of life and disease progression. Better understanding and monitoring of these comorbidities could help to improve patient management, provide more tailored therapies for different PD subgroups, and potentially also facilitate the prediction of future disease outcomes. Below, we provide a brief overview of the comorbidities considered here, their significance for quality of life in PD, and, where applicable, their associations with each other and with the future course of the disease:

- Cognitive impairment: Common in PD, ranging from mild to severe dementia. It affects daily functioning, overlaps with other comorbidities such as depression and hallucinations, and is associated with faster progression of motor symptom and poorer prognosis^18,19^.
- Dopamine dysregulation syndrome (DDS): Associated with advanced stages of PD, often characterized by compulsive use of dopaminergic medications, leading to behavioral and psychiatric symptoms. It is associated with impulse control disorders and mood disorders^20,21^.
- Depression: Common in PD, often coexisting with cognitive impairment and exacerbating motor symptoms. Early identification and treatment are essential to improve patient outcomes^17,22^.
- Hallucinations: Associated with advanced stages of PD and cognitive decline. They indicate a severe disease state and a higher risk of dementia^18,23^.
- Dyskinesia: Involuntary movements associated with long-term dopaminergic treatment, linked to advanced PD stages. Management of dyskinesia requires a balance between control of motor symptoms and reduction of involuntary movements^24,25^.
- Apathy: Often overlaps with depression and cognitive impairment, resulting in decreased motivation and engagement in daily activities^16,26^.

All of these comorbidities are associated with a reduced *quality of life* (*QoL*), which was assessed as an additional outcome variable using the Parkinson’s Disease Questionnaire-39 (PDQ-39)^24^. In addition, comorbidities may also be indicative of different PD sub-groups, such as fast vs. slow progressors (fast progressors often have higher levels of cognitive impairment, depression and dyskinesia^27–29^), or the tremor-dominant, mixed, and akinetic-rigid subtypes (tremor-dominant cases have a lower prevalence of dementia compared to akinetic-rigid subtypes, which are more prone to rapid cognitive decline and depression^30^). Although we did not have sufficient statistical power to evaluate a machine learning-based 3-group categorization of the tremor-dominant, mixed, and akinetic-rigid subtypes, we cross-validated binary classification models to distinguish between fast and slow progressors (top and bottom quartiles of patients in terms of average annual MDS-UPDRS III motor score change).

In general, classifying comorbidities and non-motor symptoms in PD is challenging due to their complex and heterogeneous nature. As these multifactorial outcomes are difficult to characterize using a single data source and mostly not directly related to gait alterations, we expanded our analysis to include clinical and metabolomics data as further predictors. This approach recognizes that comorbidities and non-motor symptoms in PD, such as cognitive impairment, depression, autonomic symptoms, and sensory impairment, may not directly manifest in motor symptoms detectable by gait analysis. Similarly, the assessment of disease progression subgroups may reveal greater differences in their metabolomic profiles that are directly related to disease mechanisms or in their clinical features that are relevant to early prodromal stages, rather than changes in gait characteristics that are more likely to reflect later, downstream consequences of the disease. By incorporating non-motor clinical data (see “Clinical data” section in the Methods part) and blood metabolomics data (biochemical markers), we can provide a more comprehensive assessment of PD progression and current non-motor outcomes. Comparing and integrating these diverse data sources allows for a thorough exploration of biological and clinical interactions, revealing subtle patterns and associations that a single-source analysis might miss. This multimodal approach aims to increase model accuracy and robustness and offer insights into the value of different data sources for detecting comorbidities and PD progression subgroups.

#### Comparative detection of comorbidities, non-motor symptoms and progression subgroups using single data sources

The ability to detect comorbidities, non-motor symptoms and progression subgroups in PD was first assessed using the three distinct data sources separately: digital gait biomarker data, non-motor clinical variables, and blood metabolomics data. For gait biomarker data, time series features from the raw gait data were used due to their higher predictive performance in the previous analyses discussed above. All models were trained using the XGB algorithm and evaluated with the same 10-fold CV framework, calculating median AUC scores for each outcome and input data type (Table 4).

**Table 4:** Cross-validated performance for predicting comorbidities, non-motor outcomes, and progression rate subgroups

Outcome/median AUC (mad)	Gait data	Clinical data	Metabolomics data
Cognitive impairment (MoCA)	0.664 (0.14)	0.759 (0.14)	^a^0.788 (0.08)
Dopamine dysregulation syndrome	0.688 (0.19)	^a^0.714 (0.32)	0.676 (0.34)
Depression (BDI)	^a^0.783 (0.14)	0.764 (0.17)	0.647 (0.11)
Hallucinations	0.673 (0.17)	0.750 (0.31)	^a^0.785 (0.08)
Dyskinesias	0.637 (0.20)	^a^0.917 (0.12)	0.667 (0.17)
Apathy (Starkstein scale)	0.598 (0.19)	^a^0.616 (0.15)	0.524 (0.13)
Quality of life (PDQ-39)	0.647 (0.17)	0.600 (0.08)	^a^0.676 (0.21)
Progression rate	0.633 (0.13)	^a^0.728 (0.13)	0.717 (0.22)

Cross-validated performance for predicting comorbidities, non-motor outcomes, and progression rate subgroups (left column) in Parkinson’s disease patients using either the time series features derived from the gait data (column 2), clinical variables (using only the non-motor features from the “Clinical data” section in the Methods part; column 3) or blood metabolomics data (column 4) as input. Quantitative outcome scores were binarized using a median threshold to obtain comparable AUC scores across different types of outcomes (for the progression rate outcome only, the fast and slow progressor subgroups were defined as the upper and lower quartiles, respectively, of the average annual change in the MDS-UPDRS III motor score, consistent with previous studies^27^). The extreme gradient boosting (XGB) algorithm was used for prediction and 10-fold cross-validation was applied. The presented scores represent the median area under the Receiver Operating Characteristic Curve (AUC) across the cross-validation cycles and their median deviation (mad).

^a^The highest median AUC achieved for each outcome.

As expected, predicting heterogeneous comorbidities and non-motor outcomes in PD as well as progression subgroups proved more challenging than estimating diagnostic, severity, and gait-related outcomes. Performance varied significantly depending on the data type and outcome, with median AUC scores up to 92% for predicting dyskinesia but low predictivity for apathy.

Clinical data were most effective in detecting dopamine dysregulation syndrome (MDS-UPDRS Part I, question 1.6; AUC: 71.4%), dyskinesias (MDS-UPDRS Part IV, question 1; AUC: 91.7%), and progression rate subgroups (top and bottom quartiles of mean annual change in the MDS-UPDRS III score, AUC: 72.8%) and had the highest AUC for apathy (61.6%), although apathy was generally difficult to detect. Metabolomics data showed superior predictive capabilities for mental and quality-of-life outcomes, including mild cognitive impairment (MoCA score < 26; AUC: 78.8%), hallucinations (MDS-UPDRS Part I, question 1.2; AUC: 78.5%), and general quality of life (PDQ-39 score median split; AUC: 67.6%).

Gait data, while less predictive for comorbidities than for disease symptom detection and motor severity outcomes, provided the highest AUC (78.3%) for depression (Beck Depression Inventory, BDI-I^30^,). This is in line with previously reported associations between depressive symptoms and quantitative gait dysfunction in the elderly^31^. Overall, gait data yielded median AUC scores between 60% to 78% for comorbidities, competitive with other approaches, indicating significant discriminative information.

These results show that while detection of non-motor outcomes and comorbidities is feasible to a limited extent with single data sources, the significant predictive power achieved by multiple diverse data sources across most of the outcomes suggests that an integrated, multimodal data analysis approach could further improve predictive accuracy. Consequently, further investigations into integrative predictive modeling using the combined data sources were conducted (see below).

#### Multimodal integrative prediction of comorbidities, non-motor symptoms, and fast vs. slow progression

To exploit the synergies of different data modalities, we combined features from all three input types: gait data (time series features), clinical variables, and metabolomics data. To ensure that these features were on the same scale, they were standardized within each fold of the cross-validation by subtracting the mean and dividing by the standard deviation. As the original features from different data types differ not only in their scales but also in their distribution characteristics, we address potential limitations associated with this integration in the Discussion section part on study limitations. The same machine learning and cross-validation approach used for the individual data sources was applied for the integrated analysis to ensure comparability.

**Fig. 3:**
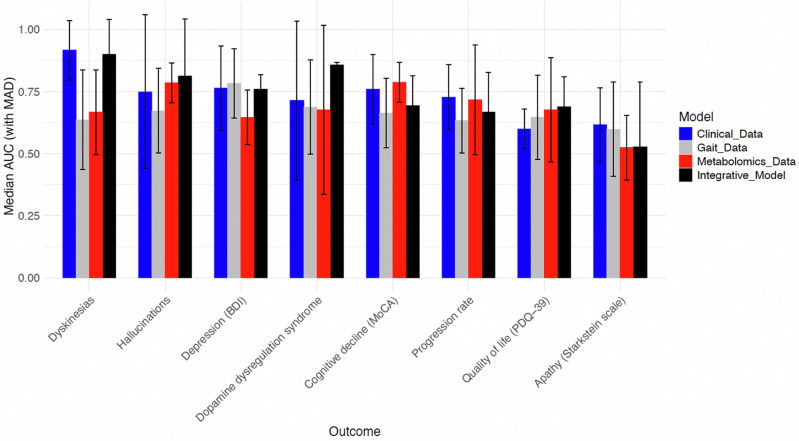
Bar plot visualization comparing the predictive performance for different PD outcomes and models using different input data. The plot compares the predictive performance in terms of median cross-validated AUC values with median absolute deviations (MAD) for the PD outcomes considered in the integrative analyses and models using different input data (Clinical Data, Gait Data, Metabolomics Data, and Integrative Model). Each outcome is represented on the horizontal axis, ordered by decreasing average AUC values across all models.

The resulting integrative models showed improved median AUC scores across multiple outcomes (Table 5 and Fig. 3). Notably, the prediction of dopamine dysregulation syndrome had a median AUC of 85.7%, considerably higher than the best individual data source (71.4%). Predictions for hallucinations and quality of life (PDQ-39) also improved, with median AUCs of 81.3% and 69.0%, compared to 78.5% and 67.6% for the best individual sources. For other outcomes, the integrative method yielded comparable performance to the average of the individual data sources, with moderate AUC values for depression (75.9%), cognitive impairment (69.4%), PDQ-39 quality of life assessment (69.0%), and progression rate subgroup (66.7%), and low performance for apathy (52.8%). Due to pronounced standard deviations, statistical significance between methodologies cannot be shown, and higher AUC values should therefore be regarded as indicative and will require further validation. Generally, while using the most informative individual data source can be advantageous for specific comorbidities, the integrative approach tends to provide competitive or better median AUCs for most outcomes.

**Table 5:** Cross-validated performance for integrative prediction of comorbidities, non-motor outcomes, and progression rate subgroups

Outcome Median AUC (mad)	Integrative Model Median AUC (mad)	Best individual data source Median AUC (mad)
Cognitive impairment (MoCA)	0.694 (0.12)	^a^0.788 (0.08)
Dopamine dysregulation syndrome	^a^0.857 (0.01)	0.714 (0.32)
Depression (BDI)	0.759 (0.06)	^a^0.783 (0.14)
Hallucinations	^a^0.813 (0.23)	0.785 (0.08)
Dyskinesias	0.901 (0.14)	^a^0.917 (0.12)
Apathy (Starkstein)	0.528 (0.26)	^a^0.616 (0.15)
Quality of life (PDQ-39)	^a^0.690 (0.12)	0.676 (0.21)
Progression rate	0.667 (0.16)	^a^0.728 (0.13)

Cross-validated performance for predicting comorbidities, non-motor outcomes, and progression rate subgroups (left column) in PD by integrating time series features from raw signal gait data, clinical variables (non-motor features from the “Clinical data” section in the Methods), and blood metabolomics data. Quantitative outcome scores were binarized using a median threshold for comparable performance scores (for the progression rate outcome only, the fast and slow progression subgroups were defined as the top and bottom quartiles, respectively, of the average annual change in the MDS-UPDRS III motor score, consistent with previous studies^27^). The extreme gradient boosting (XGB) algorithm was used for prediction with 10-fold cross-validation. The presented scores represent the median area under the Receiver Operating Characteristic Curve (AUC). Column 1 shows the different outcome measures, column 2 the median AUC scores and median absolute deviations (mad) across the cross-validation cycles for the integrative models, and column 3 the AUC scores and standard deviations for the best models based on individual data sources (see Table 4).

^a^The highest median AUC.

These results confirm the added value of comparing different data modalities and considering a multi-modal strategy in capturing the multifaceted nature of PD for detecting comorbidities. Integrating complementary data types offers a more comprehensive coverage of informative features and can therefore facilitate the machine learning based detection of multiple clinically relevant outcomes.

### Analysis of the most predictive features in the multimodal models

In addition to evaluating the predictive performance of the machine learning models using multiple data modalities, we also investigated the most predictive features identified by the SHAP value analysis. The corresponding SHAP value plots are included in the Supplementary Materials. Here, we discuss the top-ranked features for each outcome variable considered in the multimodal analyses. Because the digital gait features often lack interpretability, our discussion focuses primarily on the most predictive metabolic and clinical features for the outcomes studied.

#### Cognition (Montreal Cognitive Assessment, MoCA)

Among the most predictive features for cognitive ability as measured by the MoCA score^32^, we observed, in line with prior expectations, that age was the strongest clinical predictor (ranked third overall across the features from all data modalities, see the SHAP value plot in Supplementary Fig. 1), consistent with the well-documented association between aging and cognitive decline^33,34^. Most of the other top-ranked features in the SHAP value analysis are gait sensor features with limited interpretability (e.g., reflecting statistical moments or information-theoretic measures, such as the spectral entropy of sensor measurements). However, a few metabolites also appeared among the top-ranked predictors, including (S)-alpha-amino-omega-caprolactam (PubChem ID: 440599) as the most informative metabolite. This compound is a component in various foods and plants that, to the best of our knowledge, has not previously been associated with PD or cognitive changes before. A study of structurally similar caprolactam compounds in a mouse model of amnesia showed a significant amnesia-reversal activity for multiple of these compounds^35^, suggesting that representatives of this class of compounds may indeed affect cognitive function. However, as cognitive abilities and dietary habits can influence each other in numerous ways, an inverse causation or confounding role of diet, reflecting indirect associations to both (S)-alpha-amino-omega-caprolactam levels and cognition, cannot be excluded.

#### Dopamine dysregulation syndrome (MDS-UPDRS Part I, question 1.6)

For machine learning detection of dopamine dysregulation syndrome (DDS), most of the top predictive features were metabolite abundances, although multiple digital gait markers that lack an intuitive interpretation were also among the top-ranked features according to the SHAP analysis (see SHAP value plot in Supplementary Fig. 2). The metabolite feature with the highest SHAP value was 3-hydroxy-2-ethylpropionate (PubChem ID: 188979, also known as 2-ethylhydracrylic acid, 2-EHA), a human short-chain fatty acid derived from isoleucine metabolism. 2-EHA has been proposed both as a biomarker for isoleucine pathway defects^36^ and as a biomarker for exposure to the neurotoxin MPP^+^, as treatment of rats with the MPP+ precursor MPTP results in increased urinary 2-EHA levels^37^. Branched-chain amino acid (BCAA) metabolites, such as isoleucine, are known to affect neurotransmitter pathways, including dopamine signaling^38^, and have previously been implicated in mood disorders, such as major depression^39^ and bipolar disorder^40^. Thus, abnormal levels of 2-EHA may indicate alterations in dopamine signaling associated with either isoleucine pathway dysregulation or neurotoxin exposure. However, because variations in dietary intake can also alter the levels of BCAAs and their metabolites, such as 2-EHA, further studies are needed to determine the specific contribution of these factors to the observed alterations in 2-EHA levels in patients with DDS.

#### Beck depression inventory (BDI-I)

For the prediction of depression outcomes according to the Beck Depression Inventory scale, the highest SHAP value was achieved by a clinical predictor variable, the Sniffin’ Sticks^40^ smell test score (see Supplementary Fig. 3). Indeed, not only is loss of smell a common early comorbidity in PD, but the Sniffin’ sticks olfactory function score has previously already been found to be significantly lower in patients with depression than in controls^41^. In addition, patients with loss of smell are also more likely to have symptoms of depression, which worsens with the severity of olfactory dysfunction^42^. Although both depression and loss of smell may be influenced by confounding factors, a potential causal relationship between the two is supported by previously reported associations. Specifically, depression has been associated with a dysfunctional amygdala, which affects inhibitory projections to the olfactory bulb, and a reduced olfactory bulb volume has been observed in depression, which may impair olfactory function^43^. The second highest ranked predictor was a human metabolite, gamma-glutamylvaline (γ-EV), a dipeptide involved in glutathione metabolism^44^. Although direct studies linking γ-EV to depression have not been identified, it has been implicated in broader metabolic and inflammatory pathways that are known to influence mental health. For example, γ-EV has been reported to ameliorate TNF-α-induced vascular inflammation, which is an important factor in the pathophysiology of depression^45^. As γ-EV is found in specific foods such as legumes^46^, we note that differences in γ-EV levels between the sample groups may reflect differences in dietary habits, which could be influenced by several factors directly or indirectly associated with depression, such as socioeconomic status, access to healthy food options, stress levels, and overall lifestyle choices.

#### Hallucinations (MDS-UPDRS Part I, question 1.2)

The most predictive features for detecting the occurrence of hallucinations in PD patients include both metabolite and digital gait features (see Supplementary Fig. 4). The most informative predictor was a gait-specific feature from the “Timed Up and Go” (TUG) test, indicating altered variability of accelerometer readings in the sensor attached to the right shoe. This finding suggests that PD patients experiencing hallucinations are also more likely to have a greater gait instability.

The second ranked predictor was sucrose, commonly known as table sugar, a disaccharide composed of glucose and fructose. While differences in blood plasma sucrose levels may reflect dietary differences between groups, which could be influenced by several socioeconomic and lifestyle factors associated with a higher risk of hallucinations, a high-sucrose diet has previously been directly associated with various psychiatric phenotypes, particularly in cases with reduced expression of glyoxalase-1, an enzyme involved in the detoxification of sucrose metabolites^47^. However, reverse causation may be a more plausible explanation for the observed association, as increased consumption of sugar is not only a common feature of PD, but the consumption also increases with the occurrence of comorbidities and non-motor symptoms^48^. More focused studies are needed to confirm and better understand the potential association between dietary sucrose and hallucinations in PD.

#### Dyskinesia (MDS-UPDRS Part IV, question 1)

For the detection of dyskinesia in PD, the most informative predictor according to the SHAP value analysis was the disease duration (measured from the time of PD diagnosis by a healthcare professional to the time of assessment during the study, see SHAP value plot in Supplementary Fig. 5). This is in line with prior expectations, as it is well-established that PD patients with dyskinesia have a significantly longer disease duration than those who do not have this comorbidity^25,49^. The second highest ranking predictor was a metabolite feature, corresponding to either mannitol or sorbitol (although no disambiguation was possible based on the metabolite measurements, both of these molecules are sugar alcohols found naturally in many fruits and vegetables). Mannitol is known to temporarily disrupt the blood-brain-barrier^50^, which could affect the delivery of therapeutic agents to the brain, including PD drugs whose long-term use has been associated with dyskinesias, such as levodopa. However, similar to other comorbidities, many possible associations between dietary habits related to the levels of this metabolite and dyskinesias are conceivable (see the example of reverse causation for hallucinations discussed above), and determining potential causal mechanisms will require targeted investigations.

#### Apathy (Starkstein scale)

Of all comorbidities considered in this study, apathy was the most challenging to detect using machine learning on the available gait, molecular, and clinical data sources, achieving a maximum AUC of 61.6%. Therefore, the associated feature rankings should be interpreted with caution. The most predictive features in the integrative machine learning models include both gait and metabolomics features (see Supplementary Fig. 6). While the top-ranked gait features correspond to complex transformations of the sensor data that lack intuitive interpretation, the top predictive metabolites had known identities. The metabolite with the highest SHAP value was 1-linoleoyl-GPI (PubChem ID: 11124828). This lysophospholipid has previously been causally associated with Major Depressive Disorder (MDD) through Mendelian randomization analysis, suggesting its potential relevance to mood disorders^51^. While a potential mechanism linking 1-linoleoyl-GPI to MDD or apathy is unknown and requires further investigation, altered lipid metabolism in general has been associated with mood disorders in several studies^52–54^.

#### Quality of life in Parkinson’s disease (PDQ-39)

Although quality of life (QoL) does not reflect a specific disease symptom or comorbidity, but rather captures the patient’s overall well-being any may therefore be more difficult to relate to particular biomarkers, we still observed a higher predictive performance for the PDQ-39 QoL measure than for apathy. Correspondingly, we also identified predictors with high SHAP values in the PDQ-39 analysis, including mainly gait attributes with limited interpretability and multiple metabolites (see SHAP value plot in Supplementary Fig. 7). The most predictive metabolite was daidzein sulfate (PubChem ID: 12114465), a soy isoflavone derivative found in many soy products, which may serve as a marker for the dietary intake of these products. Soy products, which are rich in many isoflavones in addition to daidzein, have been associated with various health benefits that may indirectly improve QoL. Epidemiologic studies suggest that soy product intake may have a modest preventive role against mortality from heart disease and stomach cancer^55^, and specifically in PD, soybean intake has been proposed to have a beneficial effect on motor complications by increasing levodopa bioavailability^56^. However, because soy products contain many biomolecules that may affect health and well-being, and their intake is associated with several other lifestyle factors and dietary factors, more targeted studies are needed to confirm potential specific relationships with QoL in PD.

#### Progression rate subgroups (top and bottom quartiles of the average annual MDS-UPDRS III motor score change)

For the prediction of slow versus fast motor score progression subgroups in PD, the top-ranked variables included mostly metabolic features and a few gait sensor-derived features that lacked interpretability (see SHAP value plot in Supplementary Fig. 8). The most predictive feature was the metabolite phenylalanine, an essential amino acid that can influence neurotransmitter synthesis, potentially affecting motor function in PD patients. Specifically, phenylalanine is a precursor to tyrosine, which is subsequently converted to dopamine, a key neurotransmitter involved in motor control^57^. As the loss of dopamine-producing neurons in the midbrain is one of the main hallmarks of PD, leading to the cardinal motor symptoms of the disease, phenylalanine-derived pathways are thought to play an important role in maintaining dopamine levels in PD. Indeed, previous metabolomic analyses have already revealed alterations in phenylalanine metabolism in PD^58–60^, including changes observed in de novo patients that cannot be explained by dopaminergic treatment effects. Overall, while several confounding factors may influence the levels of phenylalanine, given its role in dopamine synthesis, its differences between fast and slow progressors may warrant further investigation.

## Discussion

Digital gait sensors represent a sensitive, easy-to-use, and largely automated method for patient monitoring, offering continuous and objective measurement of gait patterns for disease symptom diagnosis and monitoring.

Our study has shown the potential of using digital gait sensor data as a complementary biomarker tool for detecting clinically relevant outcomes in PD, specifically to facilitate the monitoring of motor score performance, the detection of gait disorders and mobility impairment, and specific comorbidities. In addition, we explored the synergies of integrating these data with complementary metabolomics and clinical data to improve accuracy for the more challenging task of classifying comorbidity and motor score progression. While digital gait sensor data alone is insufficient as a standalone biomarker, particularly for difficult-to-detect comorbidities and the more complex continuous outcome variables that have been binarized using a median threshold, its integration with other data sources can provide valuable insights and improve classification performance. By employing advanced machine learning techniques to analyze these data modalities collected from the Luxembourg Parkinson’s Study, we have observed that digital gait biomarkers can provide valuable information to distinguish between PD patients and controls, assess disease severity, and detect specific gait impairments.

Although the outcomes to be predicted in our cross-sectional study were already available from extensive clinical examinations, a machine learning analysis focused on surrogate biomarker modeling for disease monitoring was warranted for multiple reasons. First, for the assessment of motor impairment, short gait-based tests may provide a faster and less burdensome alternative to traditional clinical examinations. These short digital gait assessments could facilitate the monitoring of motor score progression by offering the possibility of reducing the frequency and duration of clinical visits, using the gait assessments as a source of information for interim assessment of disease progression. Second, while comorbidities are often studied only with a focus on future prognosis, assessing current comorbidities during clinical disease monitoring, as done in this cross-sectional study, remains a challenging and time-consuming task. This process could benefit from more objective and easily measurable biomarkers. Detection of comorbidities and other disease outcomes using digital gait data, either alone or combined with metabolomics measurements and easily obtained demographic or clinical descriptors, could provide a faster and more objective assessment. In general, we believe that digital sensor-based surrogate biomarker models for disease monitoring can offer several practical benefits for detecting outcomes that are difficult to assess in clinical examinations, including:

1. Helping to reduce frequent labor-intensive and complex clinical testing with less frequent, simpler measurements, thereby reducing healthcare costs and improving patient compliance and quality of life.
2. Supplementing or replacing largely subjective assessments with objective, data-driven assessments.
3. Enabling a more continuous monitoring to detect disease progression and specific impairments, such as gait disturbances, earlier.
4. Facilitating remote monitoring and telemedicine, increasing access to healthcare, especially for patients who have difficulty traveling to clinics.
5. Supporting disease monitoring in clinical trials and research, as detailed, objective data from digital sensors can be easily transmitted for centralized analysis.

Specifically, our results indicate that digital gait biomarkers provide significant informative value for detecting multiple diagnostic, severity and comorbidity outcomes that are commonly assessed in routine clinical practice and in PD research studies. When analyzed using current tree-based ensemble learning methods such as Extreme Gradient Boosting, gait data show robust diagnostic power for PD versus control classification, with median cross-validated AUC scores between 83% to 92% for extracted time series features. These gait features could serve as surrogate biomarkers for time-consuming and labor-intensive clinical assessments of patient mobility and specific motor and gait impairments. They have significant informative value for classifying motor severity, with median AUC scores up to 75% for distinguishing between low or high MDS-UPDRS Part III scores. This is particularly noteworthy given that gait analysis cannot measure many of the motor symptoms evaluated in the UPDRS Part III, such as speech, dysarthria, hypomimia, and tremor of upper extremities, and considering that subjective views are involved in the assignment of rating scores. For specific gait-related outcomes, such as freezing of gait (best median AUC: 92%) and occurrence of gait disorders (best median AUC: 74%), as well as general mobility assessment using the PDQ-39 mobility sub-score (best median AUC: 80%), they also provide strong diagnostic performance.

The main advantage of using digital gait sensor data for assessing these outcomes compared to classical clinical assessments lies in the ability to provide continuous and objective monitoring, potentially even in the patient’s home environment without the necessity for a hospital visit. This real-time data collection could enable earlier detection of changes in motor symptoms, allowing for more timely interventions. Although further testing and optimization of gait sensors in the home environment is needed, continuous gait monitoring has the potential to significantly reduce the frequency and duration of clinical assessments, thereby reducing the burden on both patients and healthcare providers. Such a proactive approach may help to ensure that treatment adjustments can be made promptly, based on the patient’s current condition rather than on retrospective reports or infrequent observations. Therefore, the primary added value of gait biomarkers is expected to be in the monitoring of general motor and gait impairments, offering a reliable means of tracking these impairments over time.

In addition, while reliably detecting all comorbidities, non-motor symptoms and motor score progression subgroups in PD remains challenging, for certain non-motor symptoms such as depression, there is a rationale for an association with gait impairments and our data suggests that gait sensors can provide useful information for classifying these outcomes. Previous studies have already shown that mild depressive symptoms in early PD are associated with gait impairments, including slower and more variable gait speeds^61^. Moreover, depressive symptoms and gait speed were found to be bidirectionally associated, with slower gait speeds predicting future depressive symptoms and vice versa^62^. Considering these prior studies together with the machine learning results obtained here for the Beck Depression Inventory, this suggests that gait analysis may offer valuable insights into the early detection and monitoring of at least some non-motor symptoms, such as depression.

Our study also indicated benefits of comparing and integrating gait data with complementary data modalities. While the individual data modalities—gait biomarkers, clinical features, and metabolomics—already provided valuable insights for detecting specific disease outcomes, their integration resulted in increased median AUCs for detecting the presence of multiple comorbidities, with improvements in particular for classifying outcomes such as dopamine dysregulation syndrome and hallucinations. Combining gait sensor data with a few easy-to-measure metabolites and clinical features to detect non-motor and motor outcomes could enable more objective, less time-consuming, and continuous disease monitoring, potentially facilitating more timely interventions.

Overall, this study indicates that the use of digital gait sensor data, integrated with metabolomics and clinical data, holds promise for advancing the diagnosis and severity assessment of PD and some associated comorbidities. This approach can provide an easy-to-use tool for sensitive disease monitoring, which could reduce the cost and effort associated with frequent and time-consuming clinical visits.

While these findings are encouraging, several challenges still need to be addressed before digital biomarkers can be used in routine clinical practice for PD, and it is important to consider the following study limitations to provide a balanced perspective and guide future research:

1. Sample size and cross-sectional design: The sample size in our study, while adequate for the primary analyses, is insufficient for more complex tasks such as unsupervised detection of disease subtypes. In addition, the cross-sectional design of the study limits our ability to draw conclusions about the longitudinal progression of PD.
2. Data collection and missing values: Gait measurements were taken only once during a short walking exercise of at most a few minutes’ duration. Although this approach reduces patient burden, it does not capture long-term variations in gait patterns. We note that there was a small percentage of missing values in the gait data, specifically 0.62%, with a maximum of 1.24% for any single feature. These missing values can be due to factors such as participant non-compliance, technical issues, and brief measurement interruptions. However, given the very low percentage of missingness, it is unlikely that this has significantly affected the robustness and reliability of the analyses.
3. Predictive power and use as a standalone biomarker: While digital gait sensor data showed significant potential in predicting various PD-associated outcomes, our results suggest that it is not sufficient as a standalone biomarker for most tasks. In particular, for difficult-to-detect comorbidities that are not directly related to gait impairment, digital gait data should only be considered as a complementary source of information rather than a standalone biomarker. Further optimization and validation of the models are needed before they can be applied in practice.
4. Integration of multiple data modalities: While we use standardization to adjust the scales for variables from different data modalities, the inherent differences in the distribution characteristics of these modalities can still pose challenges. Gait data, clinical variables, and metabolomics data each follow different and complex statistical distributions, which can affect the performance of machine learning algorithms. We have avoided using machine learning algorithms that require specific data distributions as input; however, the distributional differences between different input data types can still lead to biased model training, e.g., if certain data modalities dominate due to their statistical properties or larger variance. However, manual inspection of the feature selection results did not suggest the dominance of features from only a single data type. Furthermore, the integration of the gait data with metabolomics and clinical variables improved cross-validated predictions for some of the outcomes considered. We acknowledge that there may still be opportunities to further optimize and refine data integration by applying other data transformation or normalization approaches. However, because such transformations can also introduce additional noise into the data, we decided to use a simple standardization approach that already provided satisfactory cross-validation results. This approach balances the need for data consistency and model performance while minimizing the risk of introducing artifacts or bias.
5. Generalizability: The study cohort was recruited from a single center, which may limit the generalizability of the findings to broader populations. Future studies involving more diverse cohorts are needed to optimize, validate, and generalize the results before the models can be applied in practice.
6. Clinical relevance of predicted outcomes: The outcome variables considered for prediction, such as the MDS-UPDRS III motor scores, gait and motor impairment scores, comorbidity and non-motor outcome measures, and progression rate subgroups, play an important role in the monitoring of PD, but do not cover all aspects of the complexity of PD. Other potential clinically relevant disease subtypes that have been proposed in recent years^63^, but we lack sufficient sample sizes and adequate ground truth data from neuroimaging to study these recently proposed subtypes. In addition, due to the cross-sectional nature of the study, the predictive models do not provide a future prognosis, but rather assess current conditions, which limits their use in long-term disease management.
7. Technical and practical challenges: Implementation of digital gait sensor technology in routine clinical practice requires overcoming technical challenges related to data collection, transmission, and processing that go beyond the application of machine learning models and that may necessitate adaptation to the specific equipment and environment to ensure accuracy and reliability in different clinical settings. Finally, patient compliance and ease of use are critical factors that must be adequately addressed in any future study.

Future studies should focus on refining the presented disease monitoring models to address these challenges, investigating their feasibility and reliability for home assessment, and exploring their application in standard clinical practice to maximize their impact on patient care. While our study focused primarily on disease monitoring applications, it is also important to acknowledge the potential for using such integrative data for further predictive tasks, e.g., to identify additional disease subtypes beyond the fast vs. slow progression classification considered here. Multimodal approaches to disease subtyping could lead to more tailored therapeutic strategies and improve clinical care by targeting specific pathophysiological mechanisms associated with each subtype. However, detailed subtype stratification requires a larger and more diverse patient cohort, and previously proposed PD subtypes such as the “brain-first” and “body-first” classifications^63^ rely heavily on advanced imaging data such as dopamine imaging and structural MRI, which were not available for the studied cohort. In addition, further investigations should aim to differentiate PD from other neurological disorders that present with parkinsonism, such as atypical parkinsonian syndromes. Overall, while these challenges still need to be addressed, this research highlights the significant potential of digital biomarkers and multimodal data integration approaches for realizing personalized medicine for neurodegenerative diseases.

## Methods

### Study population

Participants in this study were recruited from the nationwide, monocentric, observational, longitudinal Luxembourg Parkinson’s Study^64^ under the auspices of the National Center of Excellence in Research on Parkinson’s Disease (NCER-PD). All participants gave written informed consent, and the study received a positive opinion from the National Research Ethics Committee (CNER ref: 201407/13:), ensuring compliance with all relevant ethical regulations. All research involving human research participants, material, or data were performed in accordance with the Declaration of Helsinki. To analyze cross-sectional gait changes associated with PD, digital gait sensor data were collected from 162 PD patients and 129 controls participating in the Luxembourg Parkinson’s Study. Table 6 presents relevant baseline characteristics for both PD patients and controls derived from this data set.

**Table 6:** Overview of baseline characteristics of the cohort

Characteristic	PD	Controls	*P*-value (PD vs. controls)
*N* (female/male)	162 (116/46)	129 (77/52)	0.03
Age in years at assessment (mean ± stddev.)	64.3 ± 10.7	58.5 ± 12.2	2.7E-05
MDS-UPDRS III (mean ± stddev.)	28.9 ± 13.5	3.1 ± 5.2	<2.2E-16
Hoehn & Yahr (mean ± stddev.)	2.0 ± 0.5	-	-
Disease duration since initial symptom (mean ± stddev.)	12.5 ± 7.1	-	-
BMI (kg/m2) (mean ± stddev.)	27.4 ± 4.0	27.0 ± 4.7	0.42
Presence of gait disorders (yes/no)	64/98 39.5%/60.5%	4/125 3.1%/96.9%	6.7E-16
FOGQ (mean ± stddev.)	3.2 ± 3.6	0.2 ± 0.8	1.1E-11
PDQ-39 (mean ± stddev.)	21.7 ± 14.1	5.5 ± 8.1	<2.2E-16
MoCA (mean ± stddev.)	25.5 ± 3.1	27.4 ± 2.3	2.5E-09
Dopamine dysregulation syndrome (severity scale from 0 to 4, mean ± stddev.)	0.1 ± 0.4	0 ± 0.3	0.26
Hallucinations (severity scale from 0 to 4, mean ± stddev.)	0.1 ± 0.5	0 ± 0.1	0.0011
Dyskinesias (severity scale from 0 to 4, mean ± stddev.)	0.3 ± 0.8	-	-
Apathy (Starkstein) (mean ± stddev.)	13.4 ± 5.1	9.2 ± 3.9	2.7E-14

Overview of baseline characteristics of the cohort (column 1) and the differences between PD patients (column 2) and controls (column 3), as well as the nominal p-value for the significance of the difference (column 4; the two-sided Welch’s *t* test was used for quantitative data and Fisher’s Exact Test for categorical data). For characteristics with continuous values, columns 2 and 3 show the mean and standard deviation in patients and controls, respectively; whereas for categorical variables, relative numbers per group or percentages are shown. Dopamine dysregulation syndrome was assessed using MDS-UPDRS Part I, question 1.6; hallucinations using MDS-UPDRS Part I, question 1.2; and dyskinesias using MDS-UPDRS Part IV, question 1.

#### PD diagnosis and monitoring

PD was diagnosed according to the United Kingdom Parkinson’s Disease Society Brain Bank (UKPDSBB) criteria^65^ and we use the term diagnosis throughout this manuscript to refer to the diagnostic status (PD or control) at the time of the baseline clinical assessment. Controls were included based on the following criteria: no evidence of neurodegenerative disease by clinical assessment and available imaging; age over 18 years; no current pregnancy or active cancer. Gait sensor data were used to predict the diagnostic status of PD vs. control against these gold standard criteria using cross-validation. PD and associated co-morbidities were monitored using the Unified Parkinson’s Disease Rating Scale by the Movement Disorder Society (MDS-UPDRS)^66^ and other common clinical assessments for PD, including the Parkinson’s Disease Questionnaire (PDQ-39)^67^, the Hoehn & Yahr scale^68^, the Freezing of Gait Questionnaire (FOGQ)^69^, the Montreal Cognitive Assessment (MoCA)^32^, the Starkstein scale for apathy^16^, and the Beck Depression Inventory (BDI-I)^17^. While diagnostic status, MDS-UPDRS III motor score, and gait and mobility score-related outcomes were predicted using digital gait data alone, for the more challenging tasks of predicting comorbidities, non-motor outcomes, and disease progression subgroups, we additionally built predictive models combining the gait data with clinical and metabolomics data (see “Data collection” section below).

### Data collection

#### Gait data

Gait data were collected using the eGaIT (embedded Gait Analysis using Intelligent Technologies) system^17^ with acceleration sensors attached to the shoes for 291 subjects of the Luxembourgish Parkinson’s Disease Study, including 162 PD patients and 129 controls. Measurements were made during as part of a single clinical visit during short walking exercises lasting a few minutes at most (no longitudinal measurements across multiple visits were performed in this study). Specifically, functional gait performance and mobility was evaluated using the “Timed Up and Go” (TUG) assessment^70^. Briefly, this test involves an individual standing from a seated position, walking 3.5 meters, turning around, returning to the chair, and sitting down. During the assessment, the participant is wearing the eGaIT digital sensor system on the shoes, continuously collecting gyroscope and accelerometer data to compute standardized gait parameters. In addition to this single-task assessment, the participants also performed the TUG test in parallel with further tasks, such as carrying a glass of water (“TUG Dual Task”) during the walk at either fast speed or at their preferred speed (“Preferred Dual Task”). These dual task tests are designed to evaluate the individual’s ability to handle tasks that require both cognitive and physical engagement simultaneously. Previous studies have indicated that the dual-task versions of the TUG can be more predictive of certain age- or disease-related impairments and outcomes of interest, such as fall risk, compared to the single-task version^71–73^. More detailed descriptions of the collected gait parameters and the derived features are provided in the Supplemental Material. For the data analysis, both time series features derived from the measured raw signals (spatial coordinates over time) and the extracted gait parameters for all tasks (considered as separate features) were used as input. The extracted gait parameters reflect quantitative features of the human gait, such as the mean and standard deviation of the stride length, the gait speed, or the duration of each stride. They have been computed from the raw gait sensor measurements using proprietary software by the company Portabiles^69^ (see the detailed list of gait features in Supplementary Table 1). By contrast, the time series features are less interpretable but more comprehensive. They include generic quantitative characteristics of longitudinal spatial measurement data, such as statistical moments (mean, standard deviation, skewness, kurtosis), distributional characteristics captured through quantiles and range, autocorrelation, cross-correlations, zero-crossing rate, and detrended fluctuation analysis. Additional features include lumpiness, stationarity, level shifts, variance changes, and information-theoretic measures, such as the spectral entropy (see Supplementary Table 2 for the complete list and brief description of all computed features).

#### Clinical data

The clinical data from the Luxembourg Parkinson’s Study included in the analysis encompasses a collection of demographic variables, questionnaires, and clinical scores for motor and non-motor symptoms. It covers the baseline clinical visit of the entire Luxembourg Parkinson’s Study cohort of currently 736 patients and 855 controls, including the subset of 162 PD patients and 129 controls covered by the digital gait sensor data, and we focused only on these overlapping subjects for our integrative analyses. Importantly, the health outcome variables we considered were neither overlapping with nor related to the clinical variables used as input for the integrative machine learning analyses. Specifically, the outcome variables considered in the study include the PD vs. control diagnostic status, the motor score severity as measured by the MDS-UPDRS Part III sum score^66^, outcomes related to gait and mobility impairment, including the freezing of gait (FoG) severity score from the FOGQ questionnaire^69^, the presence/absence of general gait disorders as part of the standard clinical assessments in the Luxembourg Parkinson’s Study^10^, the mobility subscore from the PDQ39 questionnaire^16^, comorbidities and non-motor outcomes (including cognitive impairment as measured by the Montreal Cognitive Assessment (MoCA)^32^, dopamine dysregulation syndrome (MDS-UPDRS Part I, question 1.6), dyskinesia (MDS-UPDRS Part IV, question 1), depression as measured by the Beck Depression Inventory (BDI-I)^73^, hallucinations (MDS-UPDRS Part I, question 1.2), apathy as measured by the Starkstein scale^16^, and quality of life (PDQ-39)^74^), and disease progression rate subgroups (fast vs. slow progressors, defined as the patients in the top and bottom quartiles, respectively, of the mean annual change in MDS-UPDRS III motor score^27^).

As input features for the clinical and integrative machine learning analyses, we used from the clinical data only a few carefully selected variables that were distinct from the outcome variables, including age, sex, body mass index (BMI), and disease duration, as well as information from a non-motor test, the Sniffin’ Sticks test of olfactory function, which has previously been shown to have significant predictive power in discriminating between PD patients and controls^74^. Hyposmia, the reduced ability to smell and to detect odors, is a significant but not specific symptom associated with PD and the prodromal stages of the disease. It provides a sensitive and easy-to-measure early indicator that can be used alongside other clinical data to enhance the predictive power of PD diagnostic models. Finally, age and sex were investigated as potential confounders in the analyses to ensure that the results are not biased by these factors. The motivation behind this small and simple selection of only five clinical input features (age, sex, BMI, disease duration, and olfactory function) was that a surrogate biomarker signature for outcomes typically derived from conventional clinical assessments would only add value if the burden of the data collection was significantly lower compared to the standard clinical examination.

#### Metabolomics data

Blood plasma metabolomics measurements were obtained through high-resolution liquid chromatography-mass spectrometry (LC-MS) and reflects the baseline clinical visit. This involved the use of a Waters ACQUITY Ultra Performance Liquid Chromatography (UPLC) system coupled with a Thermo Scientific Q-Exactive high-resolution/accurate mass spectrometer. The spectrometer was equipped with a heated electrospray ionization (HESI-II) source and an Orbitrap mass analyzer, operated at a mass resolution of 35,000. This setup allowed for detailed profiling and quantification of metabolites present in blood plasma samples from 549 PD patients and 590 control subjects in the Luxembourg Parkinson’s Study. The dataset and experimental procedures have been described in detail in the context of a previous study^75^. A complete list of the covered metabolites, including their public database identifiers, chemical properties, and associated functional metabolite classes is provided in Supplementary Data 1.

#### Intersection set of patients

**Fig. 4:**
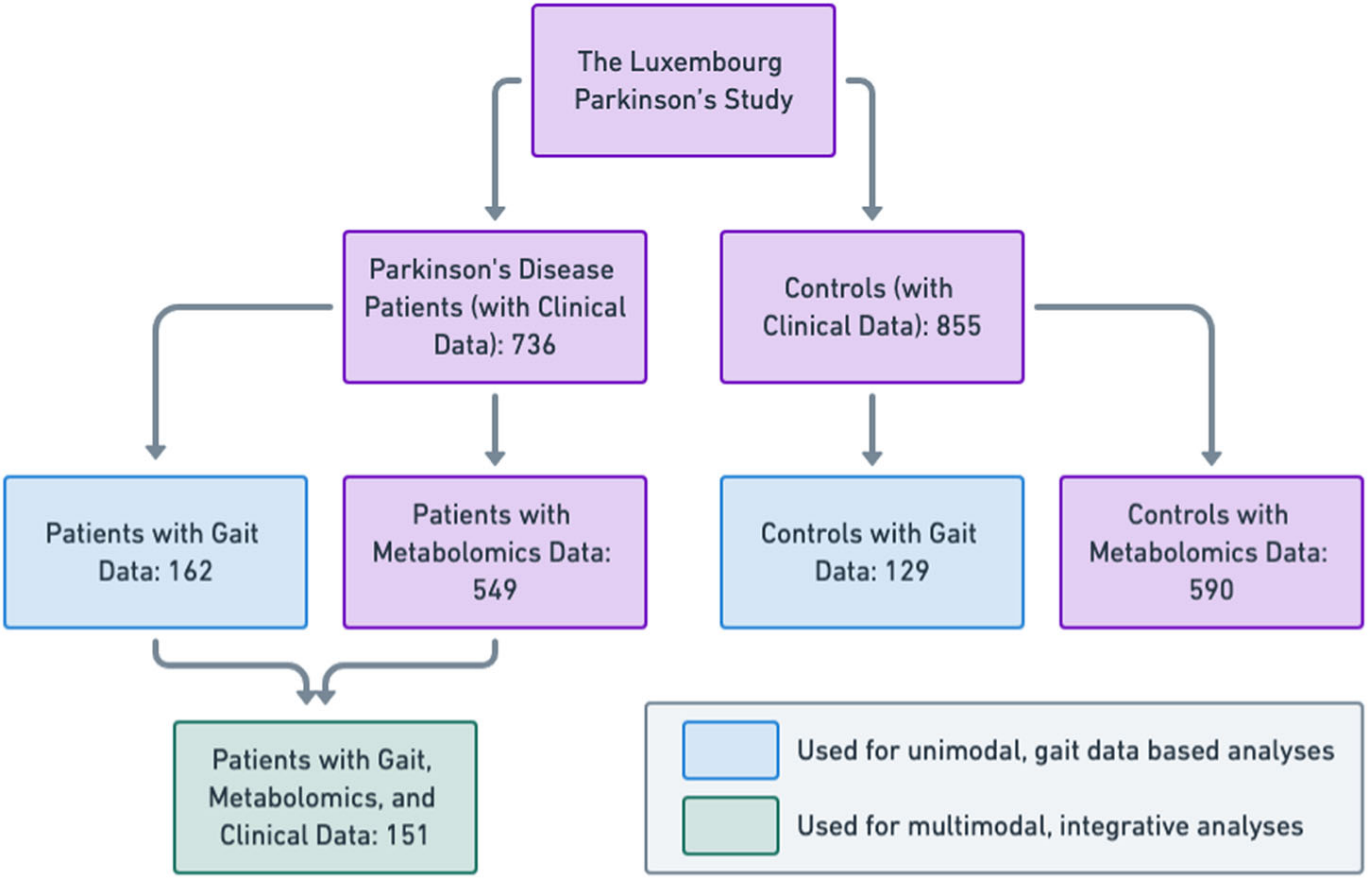
Data flow for the Luxembourg Parkinson’s Study. This figure illustrates the distribution of samples and the overlapping subsets of data types in the Luxembourg Parkinson’s Study. The study included 736 Parkinson’s disease (PD) patients and 855 controls, all of whom provided clinical data. Out of these, 162 PD patients and 129 controls had digital gait data collected. In addition, metabolomics data were available for 549 PD patients and 590 controls. For 151 PD patients, all three data types available (gait, metabolomics, and clinical data) were available and used for integrative analyses. Unimodal analyses were performed for all subjects with available gait data. The color-coded boxes indicate the specific datasets used for unimodal and multimodal analyses.

All three types of data (gait, metabolomics, and clinical) were available for 151 PD patients. This intersection set was used for multimodal, integrative machine learning analyses to detect PD comorbidities and non-motor symptoms. For motor score progression classification, a further filtering was performed to focus on patients with at least four annual clinical visits. This allowed for a robust calculation of the average annual change in MDS UPDRS III motor score and selection of the patients in the top or bottom quartile of this change, corresponding to 54 fast and 38 slow progressors, respectively. In contrast, for the unimodal machine learning predictions of PD vs. control diagnostic status, MDS-UPDRS III motor score prediction, and gait and mobility impairment detection, only digital gait sensor measurements were used (covering 162 PD patients and 129 controls), as these outcomes are expected to be more closely related to gait changes than comorbidities and non-motor symptoms. An overview of the data flow for the study is shown in Fig. 4, highlighting the samples and datasets used for unimodal and multimodal analyses.

### Data pre-processing

The data preprocessing performed in this study covered the clearing, filtering and transformation of gait sensor data, clinical data, and metabolomics measurements.

For the gait measurements, we first integrated the data across all gait tasks (see “Data collection” section) as separate features from subjects who participated in every task to enable a comprehensive analysis across multiple types of gait assessments. To impute missing values, the R software package *missForest*, an iterative method that uses random forests for imputation, was employed as part of the cross-validation process^76^. Since only 0.62% of the gait data was missing and the maximum percentage of missing values for any feature was 1.24%, none of the gait features had to be filtered out from the analysis. The method was applied after setting a random seed to ensure the reproducibility of the imputation process. Only features where more than 50% of the values were missing were completely removed from the dataset. Moreover, features that showed no variance (constant features) were identified and removed, since they provide no useful information for distinguishing between different observations in predictive modeling.

For the clinical data, in addition to the imputation and removal of constant features and variables exhibiting more than 50% missing values, categorical features containing more than ten categories were also removed to simplify the model and avoid issues with high dimensionality, which can lead to model overfitting. The remaining data was then converted to numeric format to provide a suitable input for machine learning. However, to limit the model complexity, we decided to use only a few manually selected variables from the clinical data for model building (see “Clinical data” sub-section in the “Data collection” section), and none of these features were affected by the missing value filter.

The pre-processing of the raw LC-MS metabolomics data has already been described in detail for a previous study^75^. Briefly, the pre-processing generated metabolite abundances in the form of log-transformed, batch normalized and imputed peak-area data (i.e., total ion counts, representing the integrated area-under-the-curve). For the batch normalization, experimental samples had been randomized across the batches and each metabolite’s raw values were divided by the median value of that metabolite in each batch, standardizing all batches to have a median of one. For each metabolite, the minimum value across the batches for the median-scaled data was used to impute the missing values, because missingness in this type of data is generally the result of measurements falling below the detection limit. Finally, the batch-normalized and imputed data was transformed using the natural logarithm. This was motivated by a comparison of average density estimation plots of the peak-area data before and after log transformation, suggesting that the log-transformed data better follows a normal distribution.

### Machine learning, cross-validation, and model interpretation

A schematic overview of the overall study workflow, including the different types of input data modalities used for cross-validated machine learning and the main prediction goals in terms of different outcome categories (PD vs. control diagnostic status, motor scores, gait and mobility impairments, comorbidities, non-motor symptoms, progression rates) is shown in Fig. 1. For the prediction of diagnostic status, motor scores, and gait and mobility impairments, which are closely related to changes in gait characteristics, we relied solely on the digital gait sensor data, whereas for the more challenging task of detecting comorbidities, non-motor symptoms and disease progression rate subgroups, we combined gait, omics, and clinical data to exploit the synergies of these different data modalities.

In this study, we employed a 10-fold cross-validation (CV) framework to evaluate the performance of machine learning models developed to predict clinically relevant PD outcomes using digital gait biomarkers and complementary metabolomics and clinical data. Hyperparameter optimization was performed within the CV, but only using the internal tuning procedures of the modeling approaches, otherwise default parameters were used. We did not implement additional custom parameter tuning, as our experience shows that the benefit for cross-validation performance is small, while the runtime requirements and the risk of overfitting increase. We note that the internally selected parameters may vary for each cross-validation fold. Therefore, for reproducibility purposes, rather than listing internal parameter selections for numerous cross-validation cycles and machine learning methods, we have made the code for all analyses available on GitLab (https://gitlab.com/uniluxembourg/lcsb/biomedical-data-science/bds/digital-gait-pd). We acknowledge that further optimization of the models presented in this article is still possible, but the focus of the present study was to compare the model predictivity achievable using standard machine learning implementations for different input data sources and outcome variables, rather than tuning individual models. This approach was chosen to provide a baseline understanding of model performance under default conditions, and further optimization beyond the scope of this article may be explored in future studies with larger sample sizes.

For all classification tasks, the average cross-validated area under the receiver operating characteristic curve (AUC) was used as the evaluation metric. Although the AUC could have been calculated for the entire dataset instead, we chose to use cross-validation to assess the stability and robustness of performance statistics across different data subsets. This approach helps ensure that the model’s performance is consistent and not overly dependent on any single subset of the data, by allowing us to evaluate the median absolute deviation (MAD) from the median AUC across the cross-validation cycles as a robust measure of prediction variability. The AUC statistic was preferred for measuring the performance of a binary classifier, as in contrast to other common measures, such as the accuracy, it considers both the sensitivity and specificity of the model across different threshold settings.

The machine learning models considered in this analysis included Linear SVM (Support Vector Machine), RBF (Radial Basis Function) SVM, Random Forest^77^, Stochastic Gradient Boosting^78^, Extreme Gradient Boosting (XGB)^14^ and Deep Boosting^79^. Each of these approaches has distinct characteristics and assumptions that make them suitable for different types of data and analysis needs. Logistic regression and linear SVMs are often used for binary classification tasks, providing probabilities that a given input point belongs to a certain class. Radial Basis Function (RBF) SVMs are more flexible in handling non-linear relationships via a kernel function, which implicitly maps data points to a high dimensional space. Random Forest, Stochastic Gradient Boosting and Deep Boosting are ensemble methods that aggregate the predictions of multiple decision trees to improve the model’s accuracy and robustness.

For our comparison of integrated versus individual data sources in detecting comorbidities, non-motor symptoms and disease progression rate subgroups, we focused on the XGB approach. This decision was made to avoid the extensive runtime requirements associated with applying all machine learning methods in a cross-validation for all outcomes, and because our primary goal for this part of the study was to compare the informative value of different input data types rather than different algorithms. We chose the XGB approach for this purpose because, in our initial empirical investigations, it provided a suitable trade-off between runtime performance and cross-validated AUC performance.

To aid in the interpretation of these models, we extracted SHAP (SHapley Additive exPlanations) values^15^. SHAP values help in understanding the impact of each feature on the model’s prediction for an individual sample, thereby providing insights into the behavior of the model in a transparent and interpretable manner. This is particularly important in medical applications such as ours, where understanding the decision-making process of the model can provide clinical insights and aid in further research and development of treatment strategies.

Overall, the motivation behind the outlined methodology was to provide both a thorough evaluation and optimization framework for predictive modeling and to ensure that the models are interpretable, to promote trust and deeper understanding of their practical implications in clinical settings. All analyses were implemented in the R statistical programming software (version 4.2.0^80^) and run on a physical machine (CentOS 7.9.2009, Kernel: 3.10.0-1160.25.1.el7.x86_64).

## Supplementary information

Supplemental Material

Supplementary Data 1

## Data Availability

The personal clinical, gait, and metabolomics data used for this manuscript is not publicly available as they are linked to the Luxembourg Parkinson’s Study and its internal regulations. Requests for access to the dataset can be directed to request.ncer-pd@uni.lu.

## Code availability

The analyses were implemented in R (v4.2.0) and the code is available under the MIT license in the following GitLab repository: https://gitlab.com/uniluxembourg/lcsb/biomedical-data-science/bds/digital-gait-pd.
