# Author Correction: Integrating digital gait data with metabolomics and clinical data to predict outcomes in Parkinson’s disease

**DOI:** 10.1038/s41746-025-02098-9

**Published:** 2025-11-07

**Authors:** Cyril Brzenczek, Quentin Klopfenstein, Tom Hähnel, Stefano Sapienza, Jochen Klucken, Holger Fröhlich, Enrico Glaab

**Affiliations:** 1 Biomedical Data Science Group, Luxembourg Centre for Systems Biomedicine (LCSB), University of Luxembourg, Esch-sur-Alzette, Luxembourg; 2 Department of Bioinformatics, Fraunhofer Institute for Algorithms and Scientific Computing, Sankt Augustin, Germany; 3 Department of Neurology, Medical Faculty and University Hospital Carl Gustav Carus, TUD Dresden University of Technology, Dresden, Germany; 4 Luxembourg Centre for Systems Biomedicine, University of Luxembourg, Esch-sur-Alzette, Luxembourg; 5 Luxembourg Institute of Health, Strassen, Luxembourg; 6 Centre Hospitalier de Luxembourg, Strassen, Luxembourg; 7 Bonn-Aachen International Center for IT (b-it), University of Bonn, Bonn, Germany; 8 Centre Hospitalier Emile Mayrisch, Esch-sur-Alzette, Luxembourg; 9 Laboratoire National de Santé, Dudelange, Luxembourg; 10 Association of Physiotherapists in Parkinson’s Disease Europe, Esch-sur-Alzette, Luxembourg; 11 Faculty of Science, Technology and Medicine, University of Luxembourg, Esch-sur-Alzette, Luxembourg; 12 Department of Epidemiology, CAPHRI School for Public Health and Primary Care, Maastricht University Medical Centre, Maastricht, The Netherlands; 13 Private Practice, Ettelbruck, Luxembourg; 14 Parkinson Luxembourg Association, Leudelange, Luxembourg; 15 Luxembourg Center of Neuropathology, Dudelange, Luxembourg; 16 Department of Life Sciences and Medicine, University of Luxembourg, Esch-sur-Alzette, Luxembourg; 17 Private Practice, Luxembourg, Luxembourg

**Keywords:** Parkinson's disease, Diagnostic markers

Correction to: *npj Digital Medicine* 10.1038/s41746-024-01236-z, published online 06 September 2024

In this article, NCER-PD consortium members Dr. Stefano Sapienza and Prof. Dr. Jochen Klucken meet the authorship criteria. Therefore, their names have been included in the author list. The original article has been corrected.

