# Supplementary material for: Integrating digital gait data with metabolomics and clinical data to predict outcomes in Parkinson’s disease
Source: NPJ Digit Med. 2024 Sep 6;7:235. doi: 10.1038/s41746-024-01236-z (PMC11379877; doi:10.1038/s41746-024-01236-z)
Supplement: Supplementary file 1 — Supplemental Material [file 41746_2024_1236_MOESM1_ESM.pdf]

Supplemental Material

No.	Gait feature	Unit	Description
1	Mean Max. Sensor Lift	cm	The maximum elevation of the heel from the ground during the swing phase.
2	Std Max. Sensor Lift	cm	Standard deviation of the maximum elevation of the heel during the swing phase.
3	Mean Stance Time Mean	s	Average duration from Heel Strike (HS) with the surface until Toe Off (TO).
4	Std Stance Time Mean	s	Variability of stance time duration.
5	Mean Max. Toe Clearance	cm	The maximum elevation of the toe from the ground during the swing phase.
6	Std Max. Toe Clearance	cm	Standard deviation of the maximum toe clearance during the swing phase.
7	Mean Swing Time Mean	s	Average duration from Toe Off until next Heel Strike.
8	Std Swing Time Mean	s	Variability of the swing time duration.
9	Mean Time Stamp	s	Average recorded time at which data points were collected.
10	Std Time Stamp	s	Variability of time stamps in the dataset.

11	Mean Max. Foot Clearance	cm	The maximum elevation of the foot from the ground during the swing phase.
12	Std Max. Foot Clearance	cm	Standard deviation of the maximum foot clearance during the swing phase.
13	Mean Gait Speed	m/s	Average walking speed calculated by dividing the stride length by the stride time.
14	Std Gait Speed	m/s	Standard deviation of the average walking speed.
15	Mean Turning Angle	deg	The angle between the direction of the last swing phase and the orientation of the foot in the next stance phase.
16	Std Turning Angle	deg	Standard deviation of the turning angle between swing phases.
17	Mean Toe Off Angle	deg	The angle between the heel and the surface at the beginning of the swing phase.
18	Std Toe Off Angle	deg	Standard deviation of the toe off angle at the beginning of swing phases.
19	Mean Heel Strike Angle	deg	The angle between the toes and the surface when the foot lands.
20	Std Heel Strike Angle	deg	Standard deviation of the heel strike angle when the foot lands.
21	Mean Stride Length	cm	Average distance between two consecutive Heel Strikes, representing the length of one stride.
22	Std Stride Length	cm	Variability of stride length.

23	Mean Stance Time Single Values	s	Values of stance time for each single stride.
24	Std Stance Time Single Values	s	Variability of individual stance times.
25	Mean Landing Impact	g	Maximum vertical acceleration during landing of the foot.
26	Std Landing Impact	g	Standard deviation of the landing impact.
27	Mean Max. Lateral Excursion	cm	Maximum lateral deviation of the foot in the swing phase, measured from an imaginary line between the foot's position at start and end of the swing phase.
28	Std Max. Lateral Excursion	cm	Standard deviation of the maximum lateral excursion of the foot.
29	Mean Swing Time Single Values	s	Duration of the swing phase for each individual stride.
30	Std Swing Time Single Values	s	Variability of individual swing times.
31	Mean Stride Time	s	Average duration of one stride, sum of swing and stance times.
32	Std Stride Time	s	Variability of the stride times.

Supplementary Table 1: Tabular overview of the gait-specific features derived from the digital gait sensor data, covering 32 features. Column 1 lists the types of features (including mean and standard deviation aggregations of measurements across all steps); column 2 provides information on the unit of measurement (cm = centimeter, s = second, g = gravitational acceleration; deg = angle in degrees), column 3 provides a

brief description. The features were computed from the raw gait sensor measurements using the proprietary software by the company Portabiles [5].

Category	No.	Type of Feature	Brief Explanation
**Statistical Moments**	1	Mean	The average value of the time series data.
	2	Standard Deviation	A measure of the amount of variation or dispersion of the time series data.
	3	Mode	The value that appears most frequently in the time series data.
	4	Skewness	A measure of the asymmetry of the probability distribution of the time series data.
	5	Kurtosis	A measure of the "tailedness" of the probability distribution of the time series data.
	6	Quantiles	Values that divide the time series data into equal-sized subgroups (0%-100% percentiles).
	7	Interquartile Range	The range between the 25th and 75th percentile. It measures the statistical dispersion of the data.
	8	Range	The difference between the maximum and minimum values in the time series data.
**Correlation Measures**	9	Autocorrelation	A measure of how related a variable is with a lagged version of itself.

	10	Correlations	The Pearson and Spearman correlation coefficients between different axes of the time series data.
**Energy and Frequency Features**	11	Zero Crossing Rate	The rate at which the signal changes from positive to negative or back.
	12	Spectral Entropy	A measure of the complexity or randomness of a signal, calculated using the spectral density.
	13	Teager-Kaiser Energy	An operator used to measure the instantaneous energy of a discrete time signal.
**Structural Features**	14	Detrended Fluctuation Analysis	A method for determining the statistical self-affinity of a signal.
	15	Coefficient of Variation	A standardized measure of dispersion of a probability distribution.
	16	Crossing Points	The number of times the time series crosses a defined level.
	17	Flat Spots	The longest sequence of consecutive identical values in the time series after discretization.
**Dynamic Features**	18	Lumpiness	Variance of the variances calculated over segments of the time series, indicating texture changes.
	19	Stationarity	Variance of the means calculated over segments of the time series, indicating mean shifts.

	20	Level Shift	The maximum change in the mean value of the time series over a defined window.
	21	Variance Change	The maximum change in the variance of the time series over a defined window.
	22	Time Lag	The time delay at which the autocorrelation of the signal is significant.

Supplementary Table 2: Tabular overview of the generic features computed to characterize spatial time series measurements, covering 22 features in total. Column 1 groups the features into five categories: Statistical moments, correlation measures, energy and frequency features, structural features, and dynamic features; column 2 lists the specific type of each individual feature; column 3 provides a brief description. For the detailed computation of all features, see section "Code availability".

Supplementary Data 1: Full list of metabolites studied. The full list of studied metabolites, including public database IDs, chemical properties, and associated biochemical pathways (provided as a separate dataset file for further editing and processing by the reader).

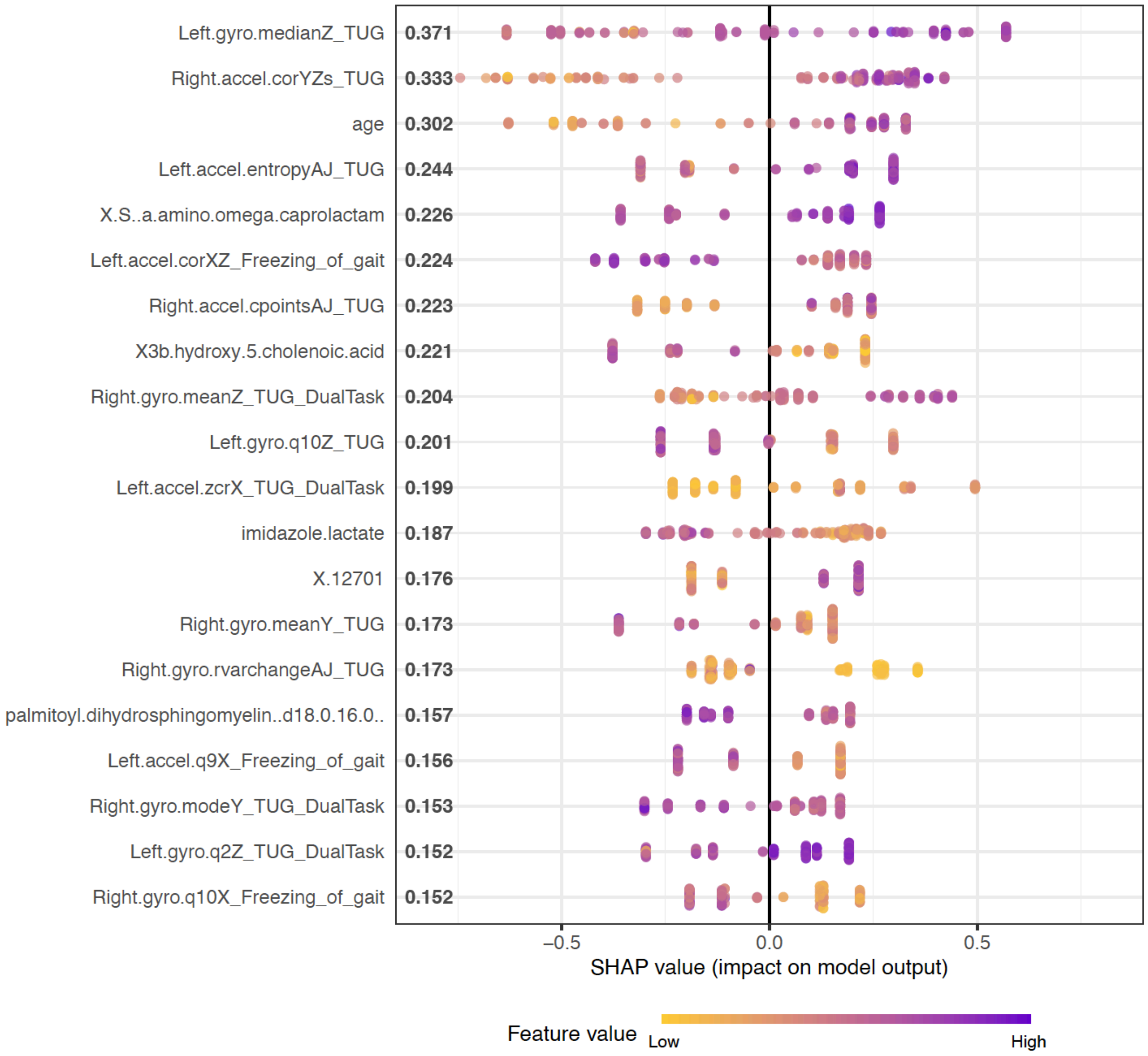

Supplementary Figure 1: SHAP value plot of the top-ranked features for predicting low vs. high Montreal Cognitive Assessment (MoCA) score outcomes using three data modalities (gait-specific digital biomarker features, clinical features, and metabolomics features) and extreme gradient boosting for machine learning. The color coding from purple to yellow represents the feature value range from low to high. The labels on the left correspond to the individual features that were most predictive in terms of the absolute SHAP value, sorted from top to bottom (corresponding absolute SHAP values are shown in bold on the left side of the plot). Feature labels starting with the label “Left” or “Right” represent digital gait sensor features measured on the left or right shoe, respectively (“gyro” stands for gyrometer; “accel” for accelerometer measurements; TUG stands for the “Timed Up and Go” walking exercise; the remaining parts of the labels reflect the feature types covered in Supplementary Table 2). Feature labels starting with the “X.” and followed only by a number rather than a metabolite name represent metabolomics features where the corresponding metabolite identity is unknown. Other features correspond to identified metabolites or clinical variables (e.g., the third top-ranked feature is the age of the patient).

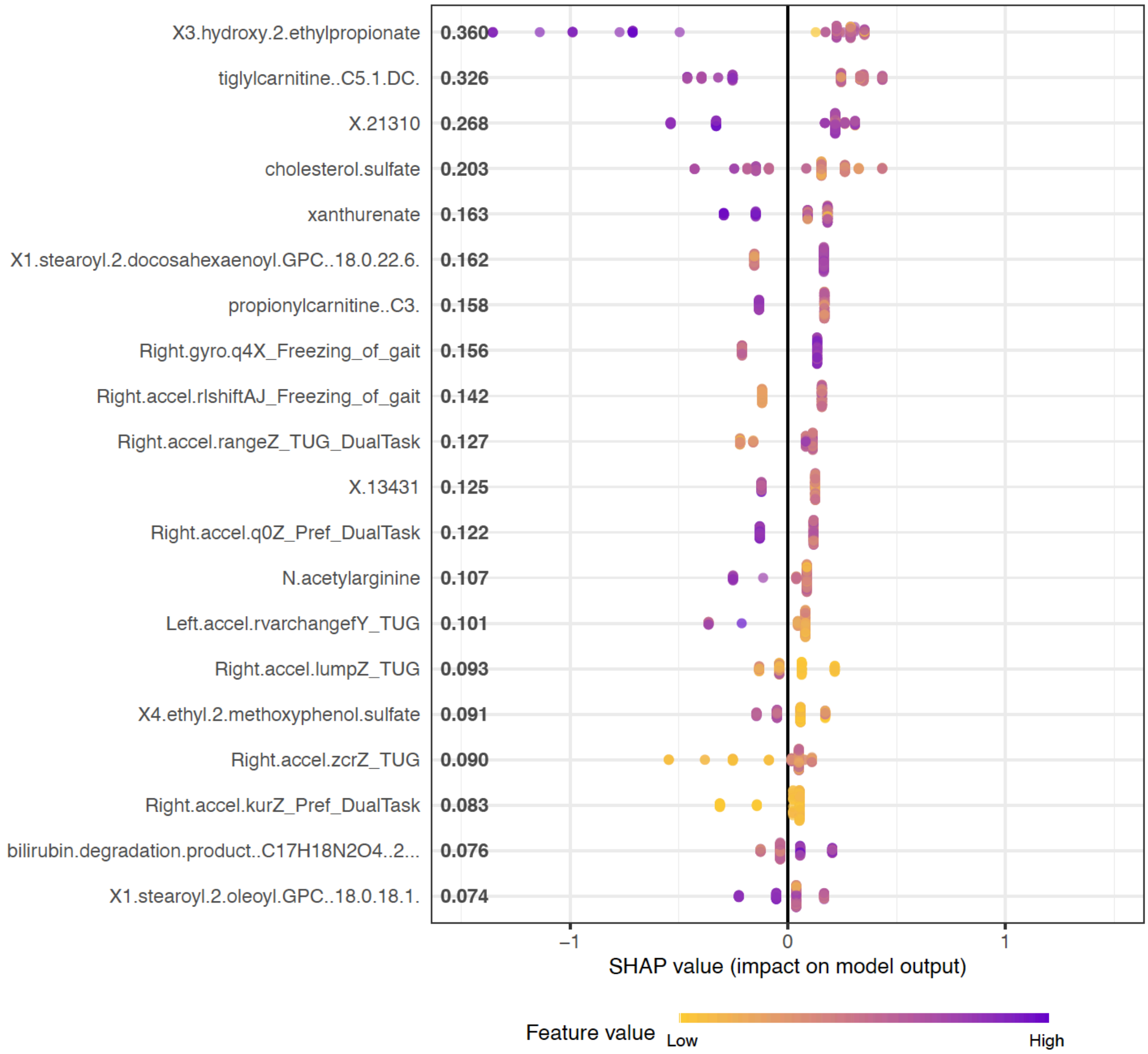

Supplementary Figure 2: SHAP value plot of the top-ranked features for detecting dopamine dysregulation syndrome (MDS-UPDRS Part I, question 1.6) using three data modalities (gait-specific digital biomarker features, clinical features, and metabolomics features) and extreme gradient boosting for machine learning. The color coding from purple to yellow represents the feature value range from low to high. The labels on the left correspond to the individual features that were most predictive in terms of the absolute SHAP value, sorted from top to bottom (corresponding absolute SHAP values are shown in bold on the left side of the plot). Feature labels starting with the label "Left" or "Right" represent digital gait sensor features measured on the left or right shoe, respectively ("gyro" stands for gyrometer; "accel" for accelerometer measurements; TUG stands for the "Timed Up and Go" walking exercise; the remaining parts of the labels reflect the feature types covered in Supplementary Table 2). Feature labels starting with the "X." and followed only by a number rather than a metabolite name represent metabolomics features where the corresponding metabolite identity is unknown. Other features correspond to identified metabolites (no clinical variables occurred among the top-ranked features for this outcome).

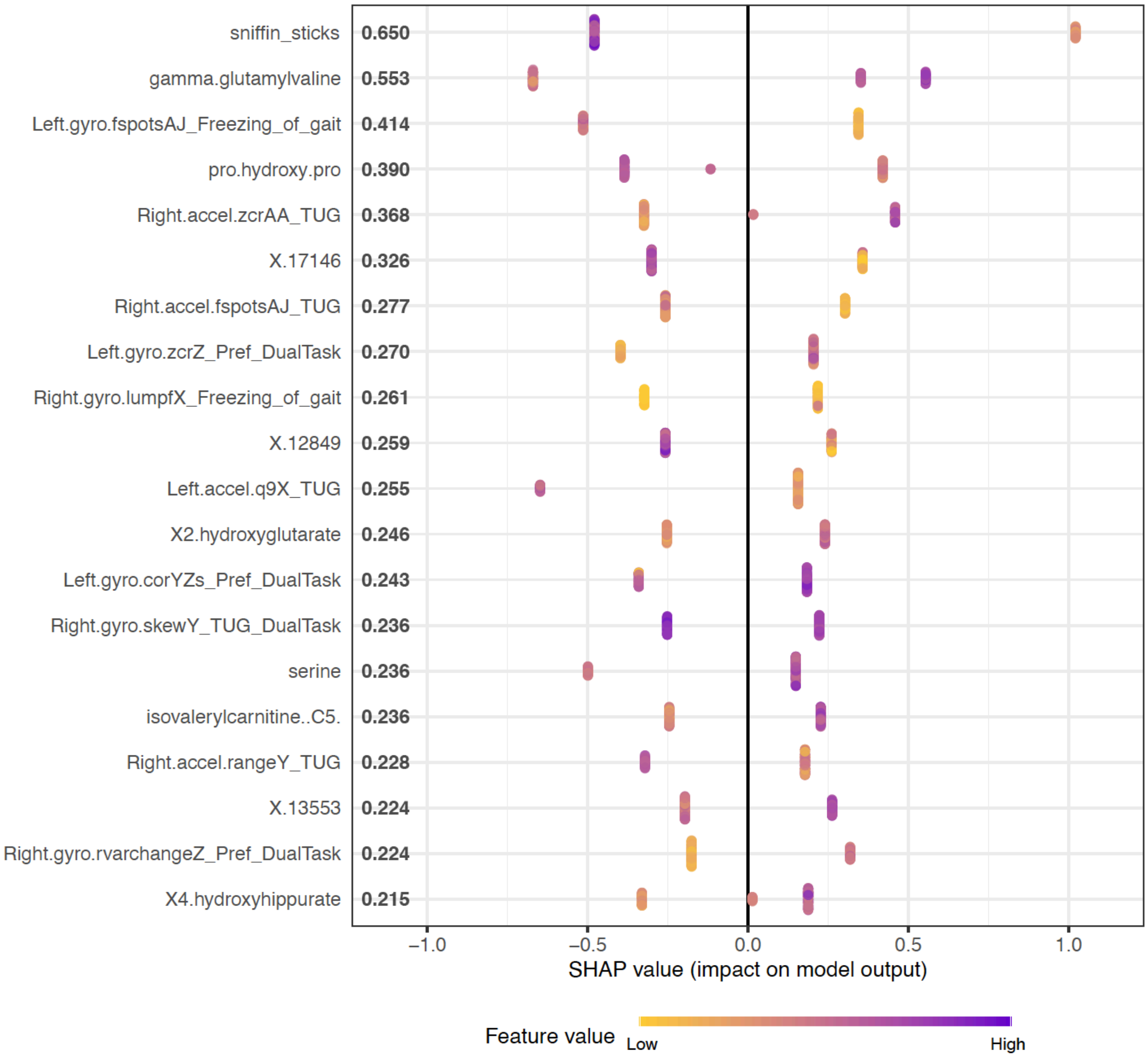

Supplementary Figure 3: SHAP value plot of the top-ranked features for predicting low vs. high Beck Depression Inventory (BDI-I) outcome scores using three data modalities (gait-specific digital biomarker features, clinical features, and metabolomics features) and extreme gradient boosting for machine learning. The color coding from purple to yellow represents the feature value range from low to high. The labels on the left correspond to the individual features that were most predictive in terms of the absolute SHAP value, sorted from top to bottom (corresponding absolute SHAP values are shown in bold on the left side of the plot). Feature labels starting with the label “Left” or “Right” represent digital gait sensor features measured on the left or right shoe, respectively (“gyro” stands for gyrometer; “accel” for accelerometer measurements; TUG stands for the “Timed Up and Go” walking exercise; the remaining parts of the labels reflect the feature types covered in Supplementary Table 2). Feature labels starting with the “X.” and followed only by a number rather than a metabolite name represent metabolomics features where the corresponding metabolite identity is unknown. Other features correspond to identified metabolites or clinical variables (e.g., the top-ranked feature is the score for the “Sniffin’ Sticks” smell test that is conducted as part of the clinical examination).

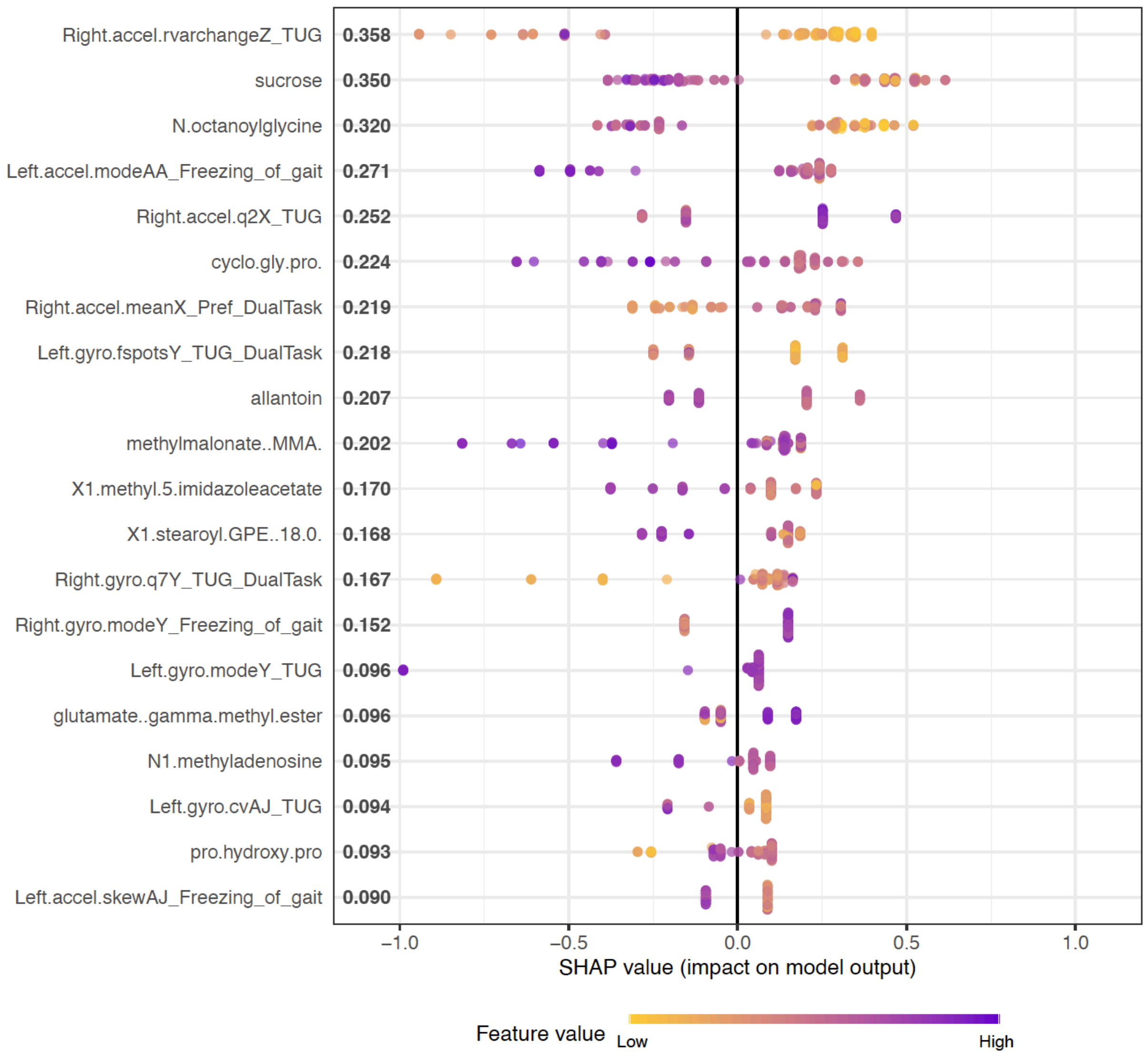

Supplementary Figure 4: SHAP value plot of the top-ranked features for detecting hallucinations (MDS-UPDRS Part I, question 1.2) using three data modalities (gait-specific digital biomarker features, clinical features, and metabolomics features) and extreme gradient boosting for machine learning. The color coding from purple to yellow represents the feature value range from low to high. The labels on the left correspond to the individual features that were most predictive in terms of the absolute SHAP value, sorted from top to bottom (corresponding absolute SHAP values are shown in bold on the left side of the plot). Feature labels starting with the label "Left" or "Right" represent digital gait sensor features measured on the left or right shoe, respectively ("gyro" stands for gyrometer; "accel" for accelerometer measurements; TUG stands for the "Timed Up and Go" walking exercise; the remaining parts of the labels reflect the feature types covered in Supplementary Table 2). Feature labels starting with the "X." and followed only by a number rather than a metabolite name represent metabolomics features where the corresponding metabolite identity is unknown. Other features correspond to identified metabolites (no clinical variables occurred among the top-ranked features for this outcome).

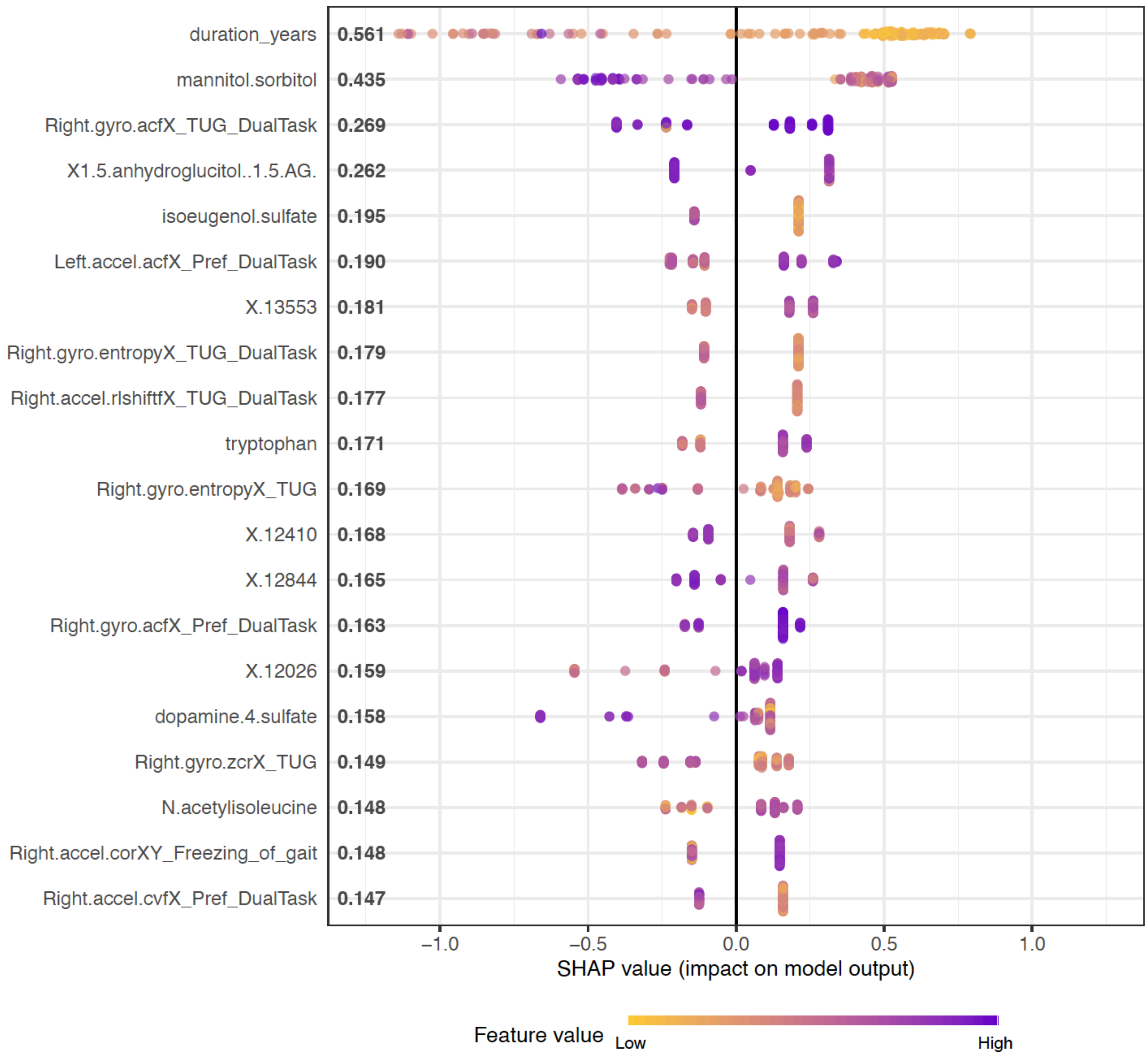

Supplementary Figure 5: SHAP value plot of the top-ranked features for detecting dyskinesias (MDS-UPDRS Part IV, question 1) using three data modalities (gait-specific digital biomarker features, clinical features, and metabolomics features) and extreme gradient boosting for machine learning. The color coding from purple to yellow represents the feature value range from low to high. The labels on the left correspond to the individual features that were most predictive in terms of the absolute SHAP value, sorted from top to bottom (corresponding absolute SHAP values are shown in bold on the left side of the plot). Feature labels starting with the label "Left" or "Right" represent digital gait sensor features measured on the left or right shoe, respectively ("gyro" stands for gyrometer; "accel" for accelerometer measurements; TUG stands for the "Timed Up and Go" walking exercise; the remaining parts of the labels reflect the feature types covered in Supplementary Table 2). Feature labels starting with the "X." and followed only by a number rather than a metabolite name represent metabolomics features where the corresponding metabolite identity is unknown. Other features correspond to identified metabolites or clinical variables (e.g., the top-ranked feature is the disease duration in years).

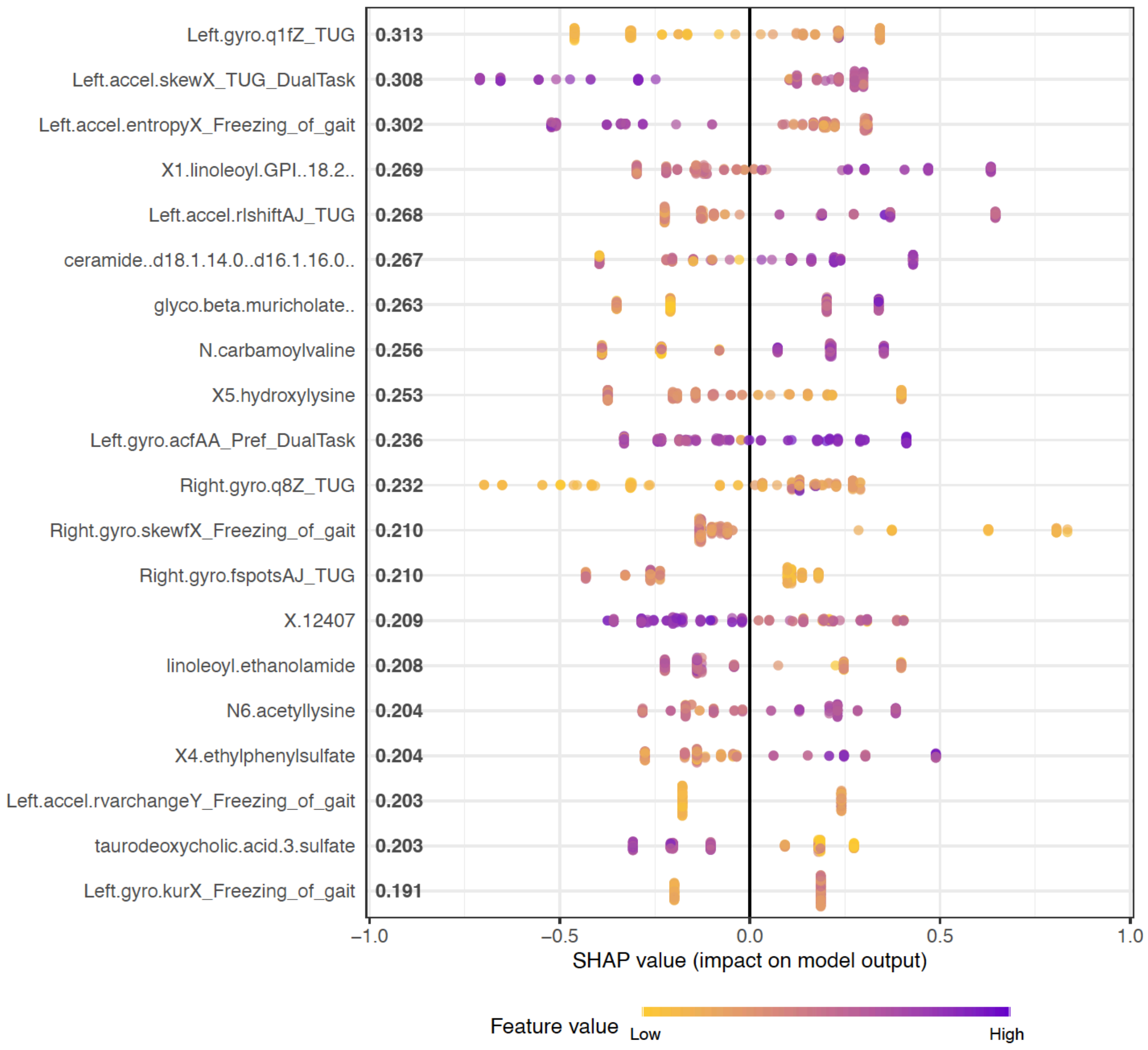

Supplementary Figure 6: SHAP value plot of the top-ranked features for predicting low vs. high apathy scores according to the Starkstein scale using three data modalities (gait-specific digital biomarker features, clinical features, and metabolomics features) and extreme gradient boosting for machine learning. The color coding from purple to yellow represents the feature value range from low to high. The labels on the left correspond to the individual features that were most predictive in terms of the absolute SHAP value, sorted from top to bottom (corresponding absolute SHAP values are shown in bold on the left side of the plot). Feature labels starting with the label "Left" or "Right" represent digital gait sensor features measured on the left or right shoe, respectively ("gyro" stands for gyrometer; "accel" for accelerometer measurements; TUG stands for the "Timed Up and Go" walking exercise; the remaining parts of the labels reflect the feature types covered in Supplementary Table 2). Feature labels starting with the "X." and followed only by a number rather than a metabolite name represent metabolomics features where the corresponding metabolite identity is unknown. Other features correspond to identified metabolites (no clinical variables occurred among the top-ranked features for this outcome).

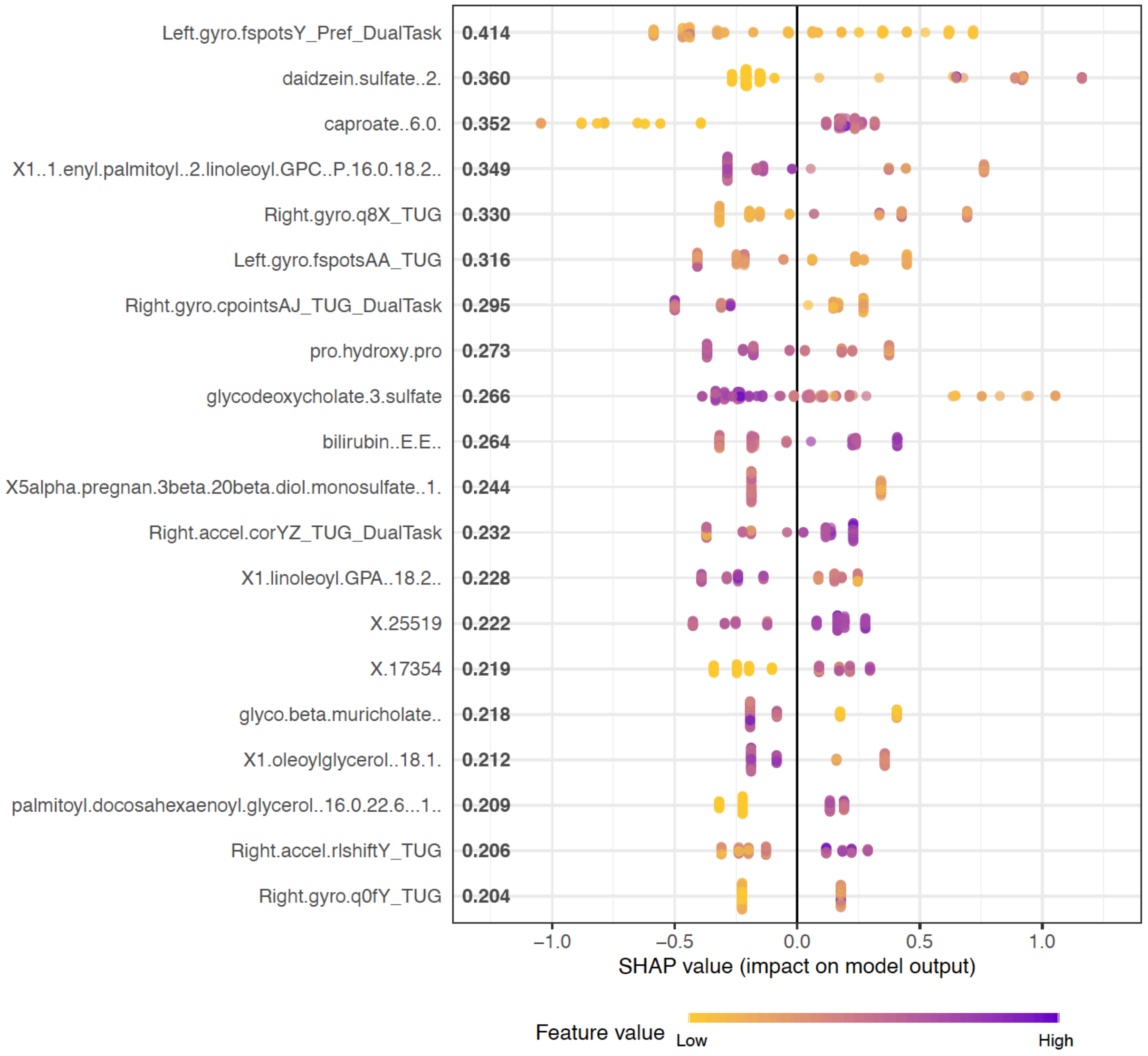

Supplementary Figure 7: SHAP value plot of the top-ranked features for predicting low vs. high quality-of-life scores in Parkinson's disease according to the PDQ-39 scale using three data modalities (gait-specific digital biomarker features, clinical features, and metabolomics features) and extreme gradient boosting for machine learning. The color coding from purple to yellow represents the feature value range from low to high. The labels on the left correspond to the individual features that were most predictive in terms of the absolute SHAP value, sorted from top to bottom (corresponding absolute SHAP values are shown in bold on the left side of the plot). Feature labels starting with the label "Left" or "Right" represent digital gait sensor features measured on the left or right shoe, respectively ("gyro" stands for gyrometer; "accel" for accelerometer measurements; TUG stands for the "Timed Up and Go" walking exercise; the remaining parts of the labels reflect the feature types covered in Supplementary Table 2). Feature labels starting with the "X." and followed only by a number rather than a metabolite name represent metabolomics features where the corresponding metabolite identity is unknown. Other features correspond to identified metabolites (no clinical variables occurred among the top-ranked features for this outcome).

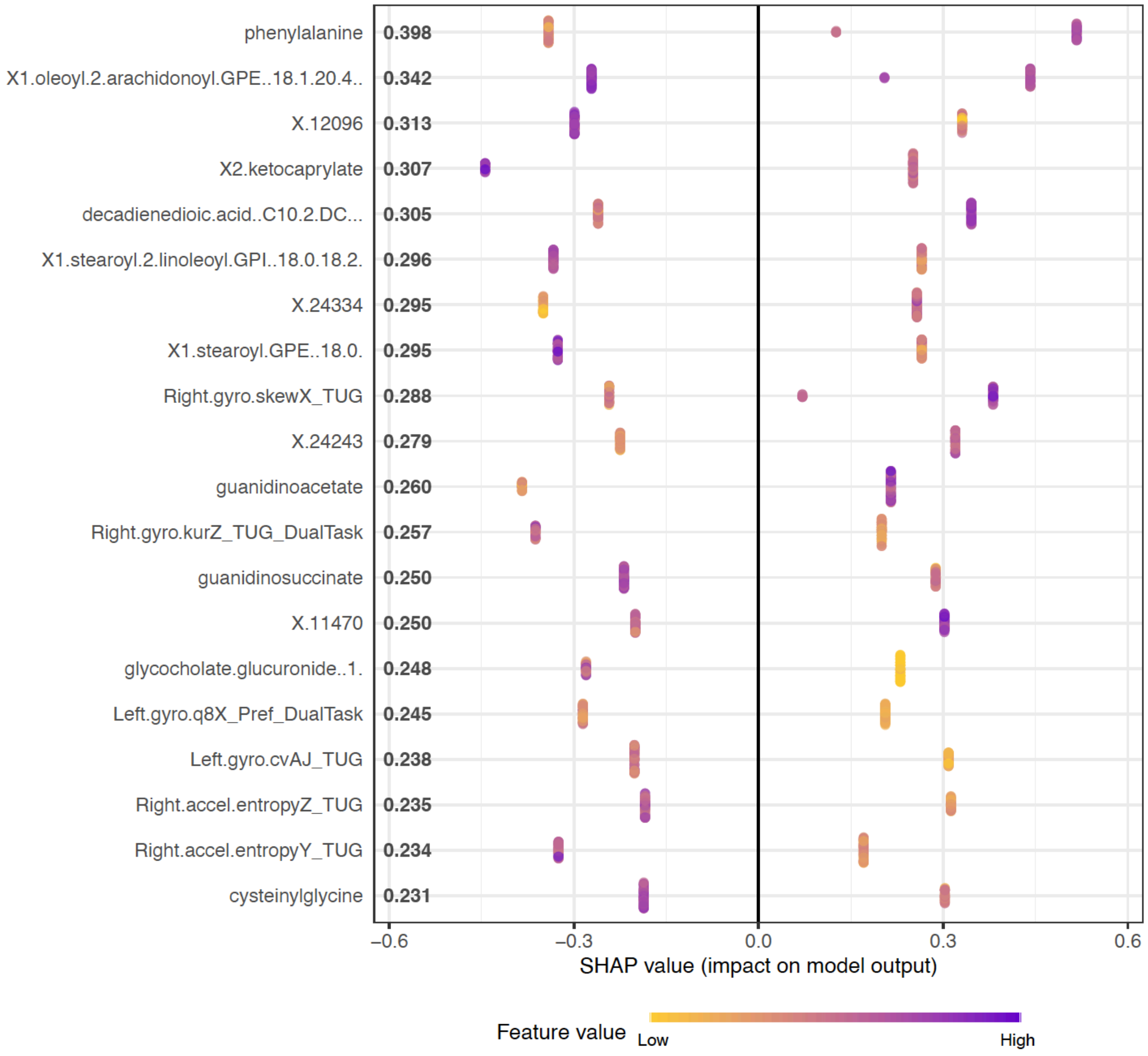

Supplementary Figure 8: SHAP value plot of the top-ranked features for detecting slow vs. fast motor score progression subgroups (defined as the patients in the top and bottom quartiles, respectively, of the average annual MDS-UPDRS III motor score change) using three data modalities (gait-specific digital biomarker features, clinical features, and metabolomics features) and extreme gradient boosting for machine learning. The color coding from purple to yellow represents the feature value range from low to high. The labels on the left correspond to the individual features that were most predictive in terms of the absolute SHAP value, sorted from top to bottom (corresponding absolute SHAP values are shown in bold on the left side of the plot). Feature labels starting with the label "Left" or "Right" represent digital gait sensor features measured on the left or right shoe, respectively ("gyro" stands for gyrometer; "accel" for accelerometer measurements; TUG stands for the "Timed Up and Go" walking exercise; the remaining parts of the labels reflect the feature types covered in Supplementary Table 2). Feature labels starting with the "X." and followed only by a number rather than a metabolite name represent metabolomics features where the corresponding metabolite identity is unknown. Other features correspond to identified metabolites (no clinical variables occurred among the top-ranked features for this outcome).
